# Nonstationary Stochastic Dynamics Underlie Spontaneous Transitions between Active and Inactive Behavioral States

**DOI:** 10.1523/ENEURO.0355-16.2017

**Published:** 2017-03-29

**Authors:** Alexandre Melanson, Jorge F. Mejias, James J. Jun, Leonard Maler, André Longtin

**Affiliations:** 1 Department of Physics, University of Ottawa, Ottawa, Ontario, Canada, K1N 6N5; 2 Department of Cellular and Molecular Medicine, University of Ottawa, Ottawa, Ontario, Canada, K1H 8M5; 3 Center for Neural Dynamics, University of Ottawa, Ottawa, Ontario, Canada, K1N 6N5; 4 Brain and Mind Research Institute, Department of Medecine, University of Ottawa, Ottawa, Ontario, Canada, K1H 8M5

**Keywords:** behavioral state transitions, bistability, computer simulations, electric fish, spontaneous movement, stochastic differential equation

## Abstract

The neural basis of spontaneous movement generation is a fascinating open question. Long-term monitoring of fish, swimming freely in a constant sensory environment, has revealed a sequence of behavioral states that alternate randomly and spontaneously between periods of activity and inactivity. We show that key dynamical features of this sequence are captured by a 1-D diffusion process evolving in a nonlinear double well energy landscape, in which a slow variable modulates the relative depth of the wells. This combination of stochasticity, nonlinearity, and nonstationary forcing correctly captures the vastly different timescales of fluctuations observed in the data (∼1 to ∼1000 s), and yields long-tailed residence time distributions (RTDs) also consistent with the data. In fact, our model provides a simple mechanism for the emergence of long-tailed distributions in spontaneous animal behavior. We interpret the stochastic variable of this dynamical model as a decision-like variable that, upon reaching a threshold, triggers the transition between states. Our main finding is thus the identification of a threshold crossing process as the mechanism governing spontaneous movement initiation and termination, and to infer the presence of underlying nonstationary agents. Another important outcome of our work is a dimensionality reduction scheme that allows similar segments of data to be grouped together. This is done by first extracting geometrical features in the dataset and then applying principal component analysis over the feature space. Our study is novel in its ability to model nonstationary behavioral data over a wide range of timescales.

## Significance Statement

Animals have the ability to initiate and terminate movement spontaneously. Given an animal moving freely in a constant sensory environment, one might expect to observe trivial behavior. Instead, spontaneous behavior is highly random and consists of a sequence of transitions between behavioral states. Identifying the intrinsic drivers of these transitions is necessary to understand more complex behaviors, and computational models are well suited to investigate the high-level processes governing the transitions. Here, we adopt a modeling approach where the neural activity that controls movement is reduced to an effective, low-dimensional process driven by noise and evolving in a nonlinear potential landscape. We show the validity of this approach in the context of spontaneous movement initiation and termination in electric fish.

## Introduction

The ability to spontaneously initiate and terminate movement is a trait shared across the animal kingdom (Kramer and McLaughlin, 2001; Maye et al., 2007; Kagaya and Takahata, 2010; Bazazi et al., 2012; Proekt et al., 2012). More generally, animals can transition spontaneously and randomly through an intricate array of behavioral states, even when their sensory environment is kept constant (Berman et al., 2014). This exemplifies how the complexity of natural animal behavior not only stems from interactions with the environment but also from an internally generated behavioral template. Yet, it is unknown what neural mechanisms trigger these behavioral state transitions and how randomness emerges in spontaneous behavior. Studies where animals are relieved of sensory stimulation are thus required to isolate and understand the internal drivers of behavior. This approach has proven useful for identifying simple principles that underlie seemingly complex behavior (Stephens et al., 2011).

In this article, we apply this approach by considering behavioral data, published by Jun et al. (2014), from electric fish swimming freely in an empty arena. While static stimuli are always present, e.g., the tank walls, the environment is devoid of any changing sensory stimuli. Like other animals and insects in these conditions, electric fish adopt an intermittent locomotion pattern where they alternate randomly between periods of rest and periods of activity (inactive and active states, respectively). Such a binary classification of behavior inevitably oversimplifies an animal’s larger behavioral repertoire. It does, however, underline key aspects of the intrinsic variability observed in animal behavior. Notably, transitions between active and inactive states seem to occur spontaneously, with the time spent in each state being highly random despite the constancy of the sensory environment. These observations imply the existence of a neural control process that triggers these transitions and that imparts a high degree of stochasticity to the behavioral data. Our goal here is to use the intermittent locomotion observed in electric fish as a means to probe the dynamical origin of spontaneous movement generation. Toward this goal, we use the Jun et al. (2014) data to constrain a low-dimensional, nonlinear stochastic model for the inferred neural control process.

More specifically, our goal is to identify a minimal set of dynamical components able to explain the core phenomenology of these data. We achieve this goal by developing a model where noisy fluctuations evolve as a diffusion process in a nonlinear, double well (i.e., bistable) potential landscape, and where, in addition, a latent, nonstationary deterministic forcing tilts the potential landscape back and forth on a slow timescale, thus modulating the rates of stochastic switching between the wells. This setup creates three interacting timescales of fluctuations: those within a single well (order of 1 s), those of the transitions between the wells (order of 10-100 s), and those of the latent variable (order 1000 s). Note that the purpose of this model is not to explain the slowest timescale but, rather, given this slow forcing, to allow the faster fluctuations to emerge freely from it. We interpret the stochastic variable of our dynamical model as a decision-like variable that, on crossing a threshold, triggers the transition between active and inactive states. We hypothesize that the latent variable represents slow neuromodulation that affect the animal’s internal states and its propensity to move.

Our study adds to the line of research that aims to understand complex phenomena with low-dimensional stochastic models (Friedrich et al., 2011). In neuroscience, such a top-down approach has been applied to model spontaneous activity in a variety of settings (Jens Prusseit, 2007; Curto et al., 2009; Deco et al., 2009; Lamouroux and Lehnertz, 2009; Mejias et al., 2010; Hindriks et al., 2011a,b). All these studies, including the present article, have the advantage of being data driven and thus require very little assumptions on the underlying biophysics. Despite the simplicity of these models, they provide powerful tools for understanding the dynamical principles that govern neural processes. Our article goes a step further in that we have developed a method to handle and model nonstationary data over long timescales.

Our main conclusion is to identify a stochastic threshold crossing process as the neural mechanism underlying the onset and offset of spontaneous movement in electric fish. In addition, we infer the existence of slow modulatory agents that impose a variable bias in the switching dynamics. The main value of our contribution is to show the applicability of a low-dimensional dynamical framework to model spontaneous natural behavior even over long periods of time where nonstationarity is involved.

## Materials and Methods

In the following, second-level subsections show the main methods and results, while third-level subsections contain the finer details and more technical information.

### Intermittency data

Intermittent locomotion, or intermittency, has been studied extensively by biologists and ecologists (Kramer and McLaughlin, 2001). These studies, however, entail monitoring unconstrained animals over long periods of time to obtain proper statistics on the movement patterns. Such experimental conditions hinder the acquisition of the concomitant neural activity, preventing any conclusions to be drawn with respect to the neural correlates of spontaneous movement. On the other hand, neuroscientists have obtained and analyzed neural data pertaining to self-initiated actions but only in the context of task-oriented experiments and only over short timescales (Libet et al., 1983; Schurger et al., 2012; Murakami et al., 2014). These experimental paradigms, therefore, do not exactly probe natural, intrinsic animal behavior.

This highlights the existence of a trade-off between observing unconstrained, freely behaving animals over timescales long enough to characterize natural intermittent behaviors, and acquiring continuous neural data required to uncover the neural basis of these behaviors. The electric fish data that we use here allow us to circumvent this problem by simultaneously providing access to a movement variable and to a proxy for high-level neural activity, namely the active sensing rate of the fish, known as the electric organ discharge rate (EODR). Both of these variables can be continuously and noninvasively recorded over long timescales (4.5-12 h). Over this period, the EODR creates a complex, bimodal time series that correlates strongly with the movement variable (Fig. 1) and that carries information on neural activity descending from the higher brain centers (Wong, 1997; Giassi et al., 2012a). An adequate model for the EODR would thus give us the ability to characterize, on a phenomenological level, the neural control process underlying spontaneous movement initiation and termination in naturally behaving fish.

**Figure:**
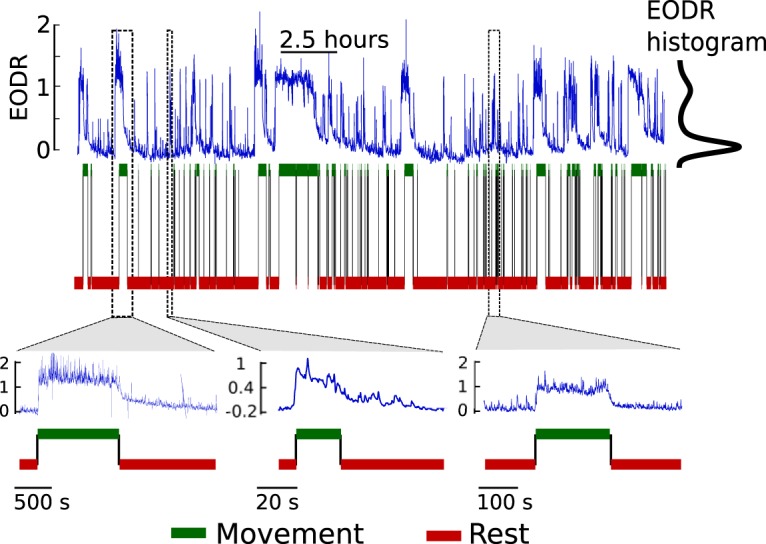
Figure 1. The EODR forms a complex, bimodally distributed time series that is highly correlated with movement. Blue traces show the EODR for Fish A, while the red and green binary traces show the movement variable, processed by a transition detection scheme (see Materials and Methods, Transition detection). To allow comparison between individuals, the EODR has been rescaled as in Jun et al. (2014), making it unitless. Insets show three examples of active states and thus reveal the diversity of activity time courses in such states.

The modeling presented in this article is based on data published by Jun et al. (2014). In the next section, we briefly describe the principles of electroreception in electric fish and then turn to the experimental paradigm and key results of the Jun et al. (2014) experiments.

### Electric fish

Electric fish possess an excitable organ, the electric organ, distributed along the length of their body that can generate an electric field around them. The spatial and temporal characteristics of this electric field depend on the specific type of fish under consideration (Bullock et al., 2005). Jun et al. (2014) used fish of the *Gymnotus* genus, which are classified as pulse-type due to the discrete nature and to the briefness of their EOD. Each pulse creates a transient, dipolar-like electric field that can be detected by the fish through electroreceptors distributed on its skin. In the absence of surrounding objects or perturbations, each pulse creates a stereotyped spatiotemporal pattern of voltage differences on the fish’s skin. This pattern is stored to memory and compared with those associated with EODs generated during normal behavior (Caputi et al., 2003). When objects are within a fish’s sensing volume, they cause deviations from this stored pattern, providing information on the type, size, and location of the object. EODs thus represent discrete sensory sampling events that allow fish to instantaneously probe their environment. This allows fish to e.g., localize and identify preys and conspecifics, as well as to navigate.

As EOD pulses are emitted in quick succession, it is often useful to consider the EODR, which provides a measure of the fish’s current level of electrosensory sampling. A high EODR corresponds to a period of heightened active sensing where the fish densely collects sensory information. Pulses are emitted at a baseline mean rate that can be modulated either spontaneously, or in response to sensory experiences. This baseline rate is established by a hindbrain pacemaker that receives input from diencephalic and medullary prepacemaker nuclei. In addition, there is evidence indicating that the EODR is strongly modulated by forebrain activity (Jun et al., 2014).

### Experiments

Experiments were conducted in a featureless, circular tank surrounded by a sensory-isolation chamber that blocked external sounds, lights, and vibrations. The only stimuli available were static: the walls and floor of the tank. Animals were kept on a 12 h light cycle, and recordings were made in the dark, during the active part of the fish’s circadian rhythm. Fish were monitored, unstimulated, for long recording sessions (4.5-12 h) while EODs were noninvasively recorded by an array of electrodes located on the periphery of the tank (Jun et al., 2012; Jun and Longtin, 2014). The number of recording sessions varied across fish. For each session, the EODR time series were obtained by smoothing the instantaneous pulse rate followed by a rescaling to allow comparison across individuals (all EODR traces shown here are thus unitless). The rescaling mapped the median EODR values during inactive and active states to 0 and 1, respectively. The time series from each sessions were then stitched together to obtain what we refer to as the pooled EODR time series.

To avoid the need for large data storage space, the movement levels of fish were obtained directly from the recorded EODs rather than from video recordings (Jun et al., 2012; Jun and Longtin, 2014). Movement information is conveyed by the EOD amplitudes at the different recording electrodes. Jun et al. (2014) showed that fluctuations of these amplitudes correlate with fish movement and can thus be converted into a movement variable. This experimental paradigm was applied to five fish of unknown sex for a total of 207 h of recording. For the analyses and modeling conducted in this article, we showcase only two of these fish, which we refer to as Fish A and Fish B. To show the general applicability of the model that we propose below, we choose these fish because they differ the most in terms of their behavioral data and thus span the largest range of behavioral types. Fish A (seven recording sessions) is an older fish that spent most of its time in the inactive state, while Fish B (eight recording sessions) is younger and much more active, with more transitions between active and inactive states. The data from the three other fish not analyzed here are qualitatively similar to those of Fish B. These fish would thus be modeled in the same way as Fish B.

These experiments revealed a strong correlation between the movement variable and the EODR: movement onset is always concomitant with a significant increases of the EODR. Moreover, the EODR tends to adopt two preferred values, as shown by the EODR histogram (Fig. 1), with increased fluctuations associated with the elevated value. By visual inspection of the EODR time series, one sees that its evolution resembles diffusional trajectories around two stable states, interrupted by sharp transitions between these states. This observation provides the foundation of the model presented below.

The fact that the EODR is bimodally distributed and that the elevated EODR value is coupled to movement allows for the definition of two behavioral attractor states, namely the active and inactive states mentioned above. In the case of these fish, active states are thus periods of movement with an elevated active sensing rate, and the transition from inactive-to-active state is concomitant with movement onset. In addition, Jun et al. (2014) report a delayed correlation on a shorter timescale, with an upward transition in EODR preceding that in movement by 1-4 s. Note that this preparatory ramp-up of the EODR is not meant to be captured by the model presented here. These observations lead to the hypothesis that a single neural control process is responsible for coupling the EODR to movement and, importantly, for triggering transitions between the behavioral attractor states (Jun et al., 2014). Because increases in EODR must be due to increased neural activity of neurons providing descending input to the pacemaker nucleus, information on the neural control process must be contained in the EODR time series. Our modeling approach and interpretations are predicated on exploiting the information content of this time series.

### Data analysis

We now turn to the details of the data analysis scheme that we develop to further probe the Jun et al. (2014) data. This is followed by the derivation and applications of the nonlinear stochastic model we propose for these data.

By visually inspecting the EODR time series, one can observe that the active states are not all alike (Fig. 1, insets). Not only do they span three orders of magnitude in terms of duration (10-1000 s approximately), but they also cover a range of different shapes; this is particularly striking in the case of Fish A. For instance, there is variability in the height of the jump around inactive-to-active transitions, which we refer to as the transition amplitude. In addition, active states show, with a varying degree, a slow tail-like decay after movement offset. We implicitly assume that if active states have similar geometrical attributes, i.e., if they “look” the same, then they are probably generated by similar neural dynamics. However, the aforementioned variability in the shape of active states leads us to hypothesize that they are not all generated by the same underlying dynamical template. To appropriately study the EODR time series, it is then necessary to group similar active states together, so as to avoid erroneously comparing states that are potentially of different neural origin, i.e., active states where the associated neural control process operates in a different dynamical regime.

To achieve this goal, we develop a dimensionality reduction scheme, similar in principle to spike sorting (Lewicki, 1998), where active states are sorted with respect to their geometrical similarities using principle components analysis (PCA). Although it is possible to apply PCA directly to the active state traces, we achieve better separation and grouping by instead quantifying active states with a small number of geometrical features, and then applying PCA on this feature space. The first step consists of breaking the EODR time series in a sequence of active and inactive states. To do so, the movement time series is piped through a transition detection scheme that assigns a time stamp to each transition (see Transition detection below). An active state is then defined as a segment of the EODR time series located between a movement onset time and a movement offset time.

**Figure 2.**
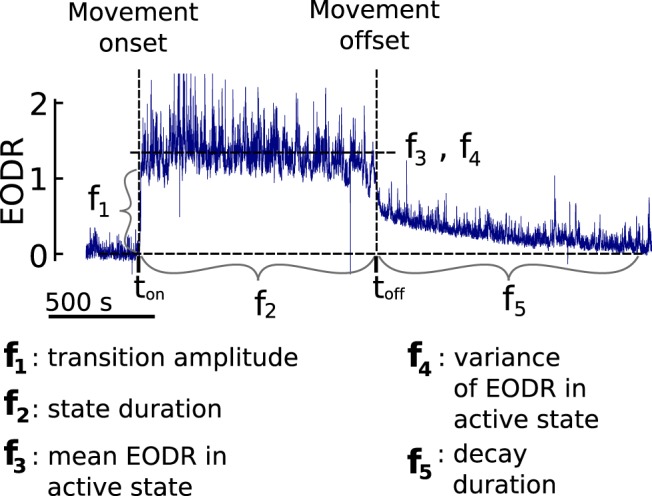
Active states are characterized by five geometrical features, *f*_1_ to *f*_5_ (see Materials and Methods, Active state features) that allow the dimensionality of the EODR time series (blue trace) to be reduced. *t_on_* and *t_off_* are the movement onset and offset times, respectively.

The second step consists of assigning a set of geometrical features for each active state (see Figure 2). Based on preliminary exploration of the data, we choose five such features, referred to as *f*_1_ to *f*_5_: the transition amplitude, the duration of the state, the average EODR during the state, the variance of the EODR during the state, and the duration of the decaying tail after movement offset. Although one might expect the transition amplitude (*f*_1_) and the average EODR (*f*_3_) to be equal, they in fact often differ. These specific features are chosen because they show significant variability across active states. Also recall that, although the EODR has been rescaled to show transitions between 0 and 1, this rescaling does not remove the variability observed in *f*_3_: to achieve this rescaling, we first obtain the median EODR for all inactive states combined, and for all active states combined, then it is these median values that are mapped to 0 and 1. The result is a rescaled EODR, the fluctuations of which yield median values in the inactive and active states of 0 and 1. The individual active states, however, still show an average value that varies around 1.

**Figure 3.**
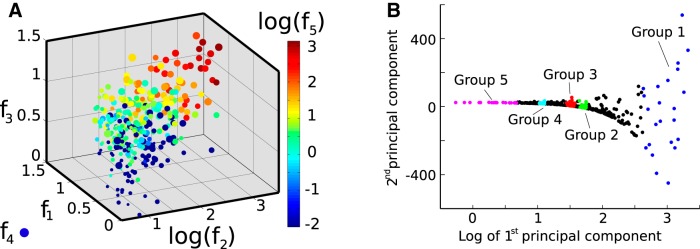
Correlations between features are revealed once the dataset is cast onto feature space. This makes PCA useful for segregating states of similar shape. ***A***, 5-D scatter plot of the active state features from Fish A. Each point corresponds to a single active state. The size of the points represent feature 4, and the colours, feature 5. ***B***, Same scatter as in ***A*** but projected onto the plane of the first two principle components. Again, each point (black and coloured) represents an active state, and colours are only added for visual representation of the different groups (colours are unrelated to those in ***A***).

With each active state represented by five coordinates, the entire time series can be visualized as a cluster of points in 5-D space, each point representing a single active state (Fig. 3*A*). This process reveals some unexpected correlations between all of the defined features. For instance, the transition amplitude (*f*_1_) correlates positively with the duration of the state (*f*_2_; see Results, Onset-triggered analysis) and with the duration of the decaying tail after movement offset (*f*_5_). This suggest that the structure and the duration of the active state is, to some extent, predetermined right from the onset. This hints at an underlying factor controlling the active states, i.e., that they are not realized from purely random processes. A positive correlation is also observed between the mean (*f*_3_) and variance (*f*_4_) of the EODR during active states. Assuming a common cause for these correlations, we apply PCA on this 5-D cluster of points in the hope of extracting some new variables (i.e., the first few principle components) along which the active states would be sorted according to their geometrical similarities. We find that, in fact, the first principle component (PC1) by itself fulfils this role satisfactorily. Although this type of sorting scheme usually leads to a search for clusters, as for spike sorting for instance, no such clusters are to be found in our case. Instead, active states are distributed continuously along the PC1 axis (Fig. 3*B*). What we observe, however, is that active states that are neighbors along this axis do look similar to one another, which is the main reason why this approach is useful here.

**Figure 4.**
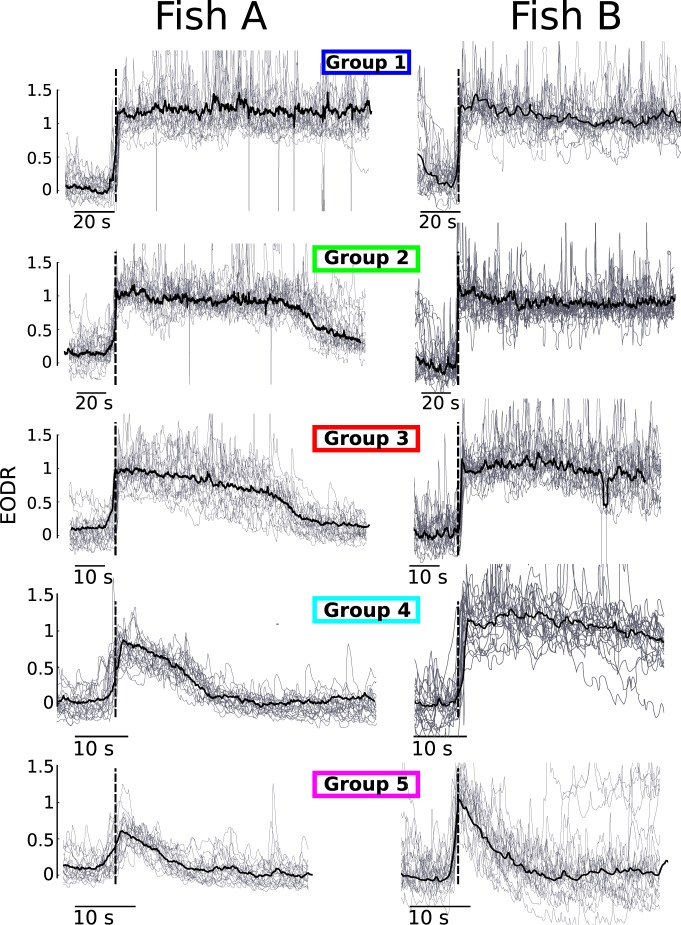
The EODR time series is populated by a heterogeneous set of active state shapes. The left panels show active states that belong to the five groups identified in Figure 3*B*, all aligned with respect to the movement onset time (dashed vertical lines). The black traces are group averages. The groups of Fish B are extracted in exactly the same way as for Fish A.

Indeed, this observation provides us with a tool to visualize and analyze the data in a new way: we can now easily plot the traces of several similar-looking active states on top of each other, and do so for the many different shapes of active states that populate the dataset. We do this by grouping together active states that are neighbors along the PC1 axis, each group centered on a different location on this axis (Fig. 3*B*). As there are no real boundaries between the active states in PC space, the number of groups we choose to define is arbitrary. Yet, we find that defining five distinct groups is enough to adequately sample the spectrum of different active state shapes found along the PC1 axis. We also find that having 20 active states per group is large enough to calculate representative average traces, yet few enough such as to avoid grouping differently shaped states together (Fig. 4). Note that, for Fish B, although the active states of groups 1-4 look similar to one another, they do indeed differ significantly in terms of their total duration and decay duration (which is not shown in Figure 4 since the traces are aligned with respect to movement onset). This similarity is largely explained by the fact that, as opposed to Fish A, the transition amplitude does not vary consistently across groups. By inspecting the left panel of Figure 4 more closely, one might also notice that the slope of the average EODR varies across groups. This means that this slope could also have been used as a geometric feature defining each active state. However, due to the high level of fluctuations in the active state, rigorously extracting this value for individual traces is problematic, and therefore, this slope was not considered in the feature space.

In summary, each group represents a stereotypical shape of active state that is found all across the dataset. For instance, for Fish A, group 1 consists of states with high values for the PC1, which corresponds to those states with long durations, long decaying tails, and higher mean values for the EODR. States that belong to group 5, on the other hand, have low PC1 values and have short durations with low transition amplitudes. Moreover, because all active states possess time stamps for movement onset and offset times, we have the freedom to analyze the groups by aligning their states with respect to either of these times. In Figure 4, for instance, the movement onset time was used as reference to align the active states. Note that, since active states within a group have slightly different durations, aligning them with respect to movement onset causes a de-synchronization around the movement offset time, i.e., the active states do not all drop off at the same time. The ability to extract and group similar active states together is essential for the modeling conducted in this article. In addition, this analysis pipeline could be applied to other types of time series and constitute a useful approach to identify and handle suspected nonstationary elements in stochastic data.

#### Transition detection

To assign a time stamp to movement onset and offset times, we start by compiling the histogram for the movement variable, which appears unambiguously bimodal, and then obtain the values for the local minimum as well as the two adjacent local maxima. To remove undesired rapid fluctuations in the movement variable, we smooth it with a moving average filter (window size, 1 s) and obtain the transition times from this filtered time series. Applying the principles of the Schmidt trigger (Fauve and Heslot, 1983), we choose the two halfway points between the local minimum and the local maxima as two distinct thresholds for detecting either movement onset or offset. An upward crossing of the upper threshold is registered as a movement onset transition, and a downward crossing of the lower threshold as a movement offset time.

#### Active state features

Here, are the details on how we define and calculate the five features used to characterize the shape of active states:

— Transition amplitude, *f*_1_. Given an inactive-to-active transition, we define two time windows, one after and one prior to the transition, both with a duration of 30 s. The transition amplitude is calculated as the difference between the average EODR within these two time windows. Note that the last 5 s of the first time window are neglected due to the presence of a short (<5 s) preparatory increase of the EODR prior to the transition.
— State duration, *f*_2_. Calculated as the difference between the movement offset and movement onset times.
— Average EODR, *f*_3_. Given by the average of the EODR over the duration of the active state.
— Variance of the EODR, *f*_4_. Given by the variance of the detrended EODR over the duration of the active state. Detrending (Matlab’s “detrend” function) is necessary since some active states show a slight downward trend.
— Decay duration, *f*_5_. Given an active-to-inactive transition, the decay duration is calculated as the time taken for the EODR to decay back to its baseline value, as calculated by the same averaging time window used for *f*_1_, prior to the movement onset.

Once PCA is performed on this feature space, we obtain the following eigenvector for the PC1: for Fish A, $PC1=0.47{\hat{f}}_{1}+0.42{\hat{f}}_{2}+0.40{\hat{f}}_{3}+0.50{\hat{f}}_{4}+0.41{\hat{f}}_{5}$, for Fish B, $PC1=0.28{\hat{f}}_{1}+0.61{\hat{f}}_{2}+0.63{\hat{f}}_{3}+0.36{\hat{f}}_{4}+0.12{\hat{f}}_{5}$, where ${\hat{f}}_{i}$ represents the unit vector for the associated axis.

#### State segregation and group definition

Once the active states have been discretized into the five features and transposed onto principle component space, we use their ordering along the PC1 axis to define groups of neighboring states that are of similar shape. Groups 1 and 5 comprise the 20 states with the highest and lowest PC1 value, respectively, while the 20 states that belong to groups 2, 3, and 4 are located above specific relative values of the PC1: 4/5, 3/5, and 1/6 of the maximum PC1 value, respectively. Those values were chosen to adequately showcase the full range of different shapes that active states adopt in the data.

### Derivation of the nonlinear stochastic model

**Figure 5.**
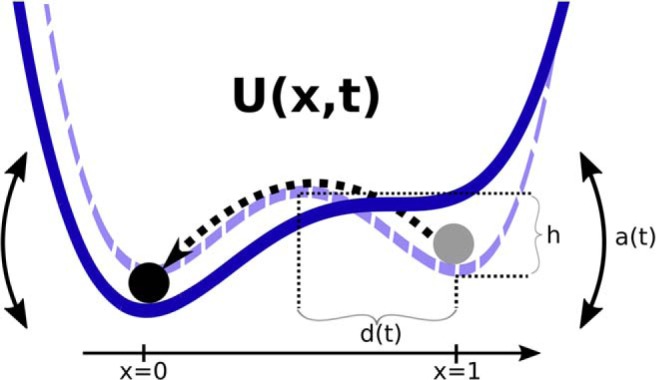
To account for key aspects of the data, we propose a dynamical system model consisting of a stochastic variable evolving in a bistable potential function that is modulated by a nonstationary, latent variable, *s*(*t*). When the tilt parameter, *a*(*t*), is zero, the potential function is symmetrical (dashed function). The potential function has two stable points: *x* = 1 represents the active state, and *x* = 0 the inactive state. For Fish A, not only does the potential function tilt back and forth, but the separation variable, *d*(*t*), is also modulated. For Fish B, the only source of nonstationarity is the tilt variable, *a*(*t*).

We outline here the details of the nonlinear stochastic model that we propose to characterize the aforementioned neural control process. Given the lack of experimental evidence available to biophysically constrain this process, it would be premature at this point to develop a detailed neural network model for it. There is rather a preliminary need to characterize the key dynamical features of the data from a phenomenological perspective. To achieve this, we propose a model with the simplest combination of dynamical components that most closely reproduces the statistics of the EODR time series. This model obeys the following stochastic differential equation:

(1) $$\frac{dx}{dt}=-\frac{\partial U(x,t)}{\partial x}+\sqrt{2D}\cdot \Gamma (t)$$

where *x* is the simulated EODR, *D* is the noise intensity, Γ(*t*) is Gaussian white noise with mean zero and autocorrelation <Γ(t)Γ(t′)>=δ(t−t′), and *U*(*x*, *t*) is a nonstationary double well potential function that can, depending on its asymmetry, give rise to bistability between two stable points separated by an unstable point (Fig. 5). It adopts the following form:

(2) $$U(x,t)=a(t)(x-0.5)+b(t){\left(x-0.5\right)}^{2}+c(t){\left(x-0.5\right)}^{4}$$

where an offset of 0.5 is introduced to have the stable points centered on 0 and 1, thereby matching the format of the EODR. Although all the parameters in Equation 2 are time dependent, the nonstationarity is in fact mediated by a single underlying variable, *s*(*t*), described below.

The second term in the right-hand side of Equation 1 is responsible for the stochastic nature of the model. Considered on its own, this term would generate a noisy, fluctuating solution, but it would lack any attractor states (i.e., fixed points). This is why we need the first term on the right-hand side of Equation 1, which is the deterministic and nonlinear component of the equation. On its own, and without nonstationary forcing, this term would establish two attractor states, i.e., bistability. This would, however, only yield a trivial solution, namely the decay to the left or right fixed point attractor, depending on whether the starting point was to the left or the right of the origin, respectively. In theory, and with infinite numerical computing accuracy, the solution would converge toward one or the other fixed point indefinitely; in practice, allowing finite precision, one simply says that the noise-free solution has reached either fixed point after a finite transient decay period. Even by adding the nonstationary forcing, the solution would merely become a binary version of the forcing, displaying transitions only when the forcing changes significantly. To display the nontrivial statistics observed in the data, the model needs the interplay between the three components: stochasticity, nonlinearity, and nonstationarity. In that case, the solution can transition randomly between the two attractor states with a rate that depends on the shape of the potential function at any given time.

The interplay between these three model components is responsible for the existence of a wide range of timescales in the solution of the model: the stochastic component creates very fast (order of 1 s) fluctuations within a single well, adding the nonlinear component allows for switching between wells on the order of 10-100 s, and the nonstationary forcing slowly modulates the transition rates between wells on the order of 1000 s.

When *a*(*t*) = 0, the potential function is symmetrical and the remaining parameters, *b*(*t*) and *c*(*t*), can be expressed as functions of the shape parameters of this symmetric double well, namely the depth of the wells with respect to the unstable point, *h*, and the separation, *d*(*t*), between the unstable point and either stable points (Fig. 5). The relations between these parameter sets are $b(t)=2h/{\left(d(t)\right)}^{2}$ and $c(t)=-h/{\left(d(t)\right)}^{4}$. When simulating the model numerically, we use these expressions as a practical way to specify the shape of the potential function *U*(*x*, *t*): we first choose a value for *h* and a time dependency for *d*(*t*), which are then used at every time step to calculate the canonical parameters *b*(*t*) and *c*(*t*). These values specify the symmetric part of *U*(*x*, *t*). Additionally, a time dependency for *a*(*t*) is prescribed and superimposed on this symmetric function, inducing a tilting of *U*(*x*, *t*) and thereby completing the definition of the deterministic part of Equation 1, for a given time step.

Based on visual inspection of the data, we choose, for Fish A, the tilt parameter, *a*(*t*), and the separation, *d*(*t*), to be both linearly dependent on a slow, latent variable, *s*(*t*), with a(t)∝−s(t) and d(t)∝s(t), see below, Integration of the stochastic differential equation for details. This configuration results in a potential function that is tilted toward the active state (*x* = 1) and has a large separation between the stable points for high values of *s*(*t*). Conversely, for low values of *s*(*t*), *U*(*x*, *t*) is tilted toward the inactive state (*x* = 0) and has a lower separation between its stable points. Also based on visual inspection of the data, we keep constant the depth, *h*, while the separation, *d*(*t*), is constant for Fish B, but remains time-dependent for Fish A.

We prescribe two different time-dependencies for the slow variable, *s*(*t*), depending on which analysis is being conducted (for details on how *s*(*t*) is specified in both cases, see below, Estimation of the latent variable). In the first case, we run Monte Carlo simulations on a short timescale around active-to-inactive transitions and thus simply prescribe a linear decay for *s*(*t*), which we refer to as *s_local_*(*t*). In the second case, simulations are performed over the timescale of the experiments (several hours), which warrants a more realistic *s*(*t*). To obtain a variable that could realistically represent the slow driving force that modulates the potential function, we apply a moving average filter with a large window to the EODR time series and we use this filtered trace as *s*(*t*). The rationale behind this method is that a smoothed version of the EODR contains the desired information on a slow timescale and thus constitutes a first estimate for the latent variable, should it exist.

Although this moving average filter will smooth out abrupt transitions, the simulations driven by this latent variable will still show transitions as abrupt as in the data, i.e, on a much faster time scale than the one on which the latent variable fluctuates. The slower rise or decay of the latent variable will, however, allow transitions to occur at a rate that is slowly modulated by this latent variable, as is observed, for instance, in the data following long active states.

Also note that by using the experimental data to infer the latent variable, and then using this latent variable to drive the model, we inevitably impart some level of correspondence between the data and the model results. This will, however, only be the case for timescales longer than the averaging window used to estimate the latent variable (∼2300 s for Fish A, ∼340 s for Fish B). Hence, out of the three timescales mentioned above, only the first two emerge directly from the model. For instance, the longest active states observed in the data (such as those shown by the black arrows in Figure 7*A*) will automatically be reproduced by the model, since they yield a very high value for *s*(*t*) when averaged out, which in turn allows a single attractor (namely, the one to the right of the origin) to exist in the model.

Our modeling framework and our interpretations are predicated on the following set of assumptions:

1. The spontaneous decision to initiate or terminate movement emerges from an open-loop subsystem, i.e., it is intrinsically generated by a high-level neural population.
2. The activity of this subsystem can be projected onto a low-dimensional manifold containing bistable attractor dynamics.
3. Neural noise causes this subsystem to randomly alternate between the two stable modes of activity, or attractor states.
4. Modulatory agents, e.g., monoamines or peptides, evolving on a slow timescale, tilt the bistable attractor landscape back and forth and thus affect the animal’s propensity to move.
5. Information on the activity of this subsystem is conveyed through the EODR. The EODR thus represents a proxy for the neural activity responsible for triggering spontaneous transitions between the active and inactive behavioral states.

Assumption 1 is plausible given that the experiments were conducted without any external cues, precluding any sensory-evoked responses. The plausibility of assumptions 2 and 3 can be established in light of the fact that (1) recurrent networks have been shown to generate bi- and multistable attractor dynamcis, in a winner-take-all fashion (Wang, 2002; Martí et al., 2008), (2) it has been suggested that neural noise can be responsible for triggering transitions between the attractor states (Martí et al., 2008), and (3) a bistable attractor network model (Wang, 2002) can be reduced to a 1-D stochastic differential equation of the same form as Equation 1 (Roxin and Ledberg, 2008). Furthermore, experiments on zebrafish have identified a clear link between neuropeptidergic modulation and arousal behavior (Prober et al., 2006; Woods et al., 2014), which supports assumption 4. Assumption 5 was discussed in the Introduction and is consistent with previous findings (Wong, 1997; Giassi et al., 2012a; Jun et al., 2014).

#### Integration of the stochastic differential equation

To integrate Equation 1, the asymmetry parameter, *a*(*t*), the separation, *d*(*t*) (or *d* =constant for Fish B), the height *h*, and the noise intensity, *D*, need to be specified. Both *a*(*t*) and *d*(*t*) are linearly rescaled versions of *s*(*t*):

(3) $$\begin{array}{l} a(t)=\frac{\left(a_{2}-a_{1}\right)}{\left(s_{max}-s_{min}\right)}\cdot (s(t)-s_{min})+a_{1}\\ d(t)=\frac{\left(d_{2}-d_{1}\right)}{\left(s_{max}-s_{min}\right)}\cdot (s(t)-s_{min})+d_{1} \end{array}$$

where *s_max_*, *s_min_*, *a*_1_, *a*_2_, *d*_1_, *d*_2_ are the extremum values of *s*(*t*), *a*(*t*), and *d*(*t*), respectively. Note that *s_max_* and *s_min_* are not free parameters, but are rather obtained from the *s*(*t*) time series. Parameter values for both fish are shown in Table 1. Parameters are obtained sequentially under various constraints that minimize the differences between simulations and experimental results. First, *d*_1_ and *d*_2_ are chosen to match the minimum and maximum transition amplitude calculated from the data (Fig. 9, groups 5 and 1, respectively). Note that assigning *d*_1_ = *d*_2_ for Fish B yields a constant *d*(*t*). Once the separation is fixed, *h* can be used to adjust the slope of the potential function between the stable and unstable point, and therefore controls the abruptness of the transitions between stable points. It is chosen such that simulated transitions are as abrupt as those observed in the data. Transitions from Fish B are slightly steeper than those of Fish A (Fig. 9), which is why we obtain a larger depth for the simulations of Fish B. *a*_1_ and *a*_2_ are chosen such that the potential function, in its most asymmetric configurations, allows states and transitions that are similar to those of the data. For instance, when *s*(*t*) adopts its highest values, the experimental data for both fish show active states that are long, with rare instances of brief inactive states (Figs. 7 and 8, top panels). This observation constrains the value of *a*_2_: when *a*(*t*) = *a*_2_, the potential function must have a deep active state well along with a shallow inactive state well. On the other hand, when *s*(*t*) adopts its lower values, this situation is reversed in the case of Fish A, while for Fish B, the EODR alternates between active and inactive states, with a bias for the inactive state (Fig. 8, upper panel). This argues for a potential function that is slightly more symmetrical than that of Fish A, which is why the value of *a*_1_ is lower for Fish B (recall that *a*(*t*) = 0 yields a symmetrical potential function). The remaining free parameter, *D*, controls the switching rate between the stable points. It is therefore obtained such that the number of transitions in the simulations matches that seen in the experimental data over the same period of time, and with the same *s*(*t*). Numerical integration is performed with the Euler–Maruyama scheme with a time step of 0.01 s.

**Table 1:** Numerical integration parameters for the models of both fish

Parameter	Fish A	Fish B
*h*	−0.08	−0.32
*d*_1_	0.3	0.5
*d*_2_	0.6	0.5
*a*_1_	0.12	0.07
*a*_2_	−0.1	−0.1
*D*	0.02	0.1

#### Estimation of the latent variable

The latent variable, *s*(*t*), is responsible for the nonstationarity imparted to the model, i.e., a constant *s*(*t*) would turn Equation 1 into a stationary stochastic process. Depending on which analysis is considered, we prescribe two different forms for *s*(*t*): it is either a slow and stochastic time series evolving over a long timescale, or a linear decay over a short timescale. The latter is referred to as *s_local_*(*t*).

For the longer simulations performed in the Results section, Onset-triggered analysis and Residence time distributions (RTDs), the stochastic differential equation (Eq. 1) is integrated over a long period of time where the latent variable, *s*(*t*), is chosen as the moving average filtered version of the EODR time series (Figs. 7 and 8, black traces). The window size for the filter is chosen as 4$\left(<T_{act.}>+<T_{inact.}>\right)$, where < *T_act_*_._ > and < *T_inact_*_._ > are the mean residence times of the active and inactive state, respectively. The window size for Fish A is 2997 s, while for Fish B it is 344 s. The window size is chosen large enough such that a change in the filtered EODR implies that the durations of the states undergo a significant, sustained deviation from their average values, which relates to the level of tilt of the potential function. The window size is also chosen such that *s*(*t*) becomes approximately constant when the true latent variable is constant, i.e., when the process is stationary (as tested with simulations).

The Monte Carlo simulations performed in Results, Offset-triggered analysis, on the other hand, are performed over only 1500 s, with a prescribed linear decay for *s_local_*(*t*). This represents a potential function, tilted toward the active state at first, that undergoes a tilting toward the inactive state at a constant rate. This rate, i.e., the slope of the linear decay of *s_local_*(*t*), is chosen as -0.036. This value is obtained under the constraint that the transient period of bistability in simulations is as long as that seen in the data (Fig. 10). A higher tilting rate [i.e., a steeper slope for *s_local_*(*t*)] would shorten this bistable period, whereas a lower rate would lengthen it. Since the influence of *s_local_*(*t*) is mediated only through linearly rescaled versions of itself, i.e., *a*(*t*) and *d*(*t*), the y-intercept of the linear profile is arbitrary.

## Results

Below, we present three distinct modeling experiments aimed at validating our proposed modeling framework. With the first two experiments, we examine trajectories of the EODR around the transitions between states. The third experiment shows how our simulations yield RTDs consistent with those seen in the data.

### Onset-triggered analysis

**Figure 6.**
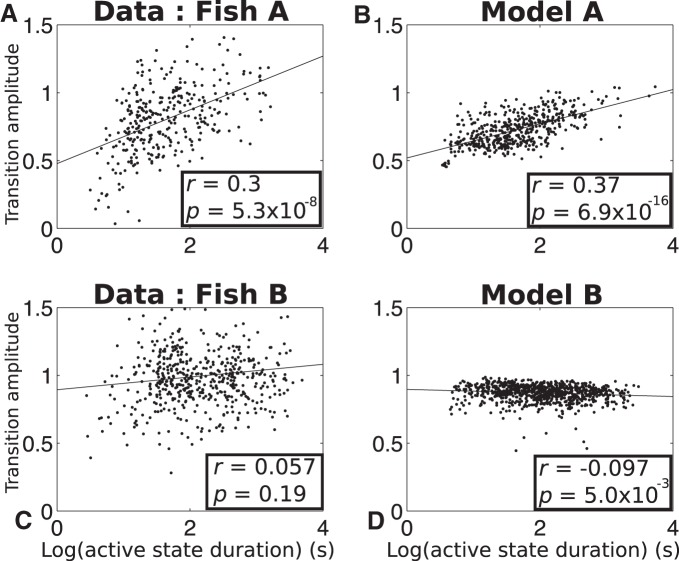
The model captures the correlation (Fish A), or lack thereof (Fish B), between the transition amplitude (*f*_1_, *y*-axes) and the duration of the active state (*f*_2_, *x*-axes). Each black dot represents an active state, projected on the *f*_1_ – *f*_2_ plane. For visual reference, a least-square line is also shown (straight black lines). The linear correlation coefficient and the *p* value for each plot are also reported as *r* and *p*, respectively.

Here, we examine how the EODR behaves around the transitions from the inactive to active state, that is, around the times of movement onset. We first investigate the correlations between the transition amplitude (*f*_1_) and the active state duration (*f*_2_) by projecting the entire feature cluster of Figure 3*A* onto the *f*_1_ – *f*_2_ plane, such as to generate 2-D scatter plots. Comparing the scatter plots for both fish (Fig. 6*A*,*C*) reveals that, for Fish A, the transition amplitude covaries with the active state duration across the dataset, with shorter states having the lowest amplitude and longer states the greatest. For Fish B, however, the transitions from inactive to active states have an amplitude that is uncorrelated with the state duration.

The unexpected variability in the dynamics of inactive to active state transitions implies important differences in neural network dynamics across individuals of the same species. This discovery invalidates blind across-individual averaging of behavioral or neural data and, as described below, requires individual-specific model choices.

**Figure 7.**
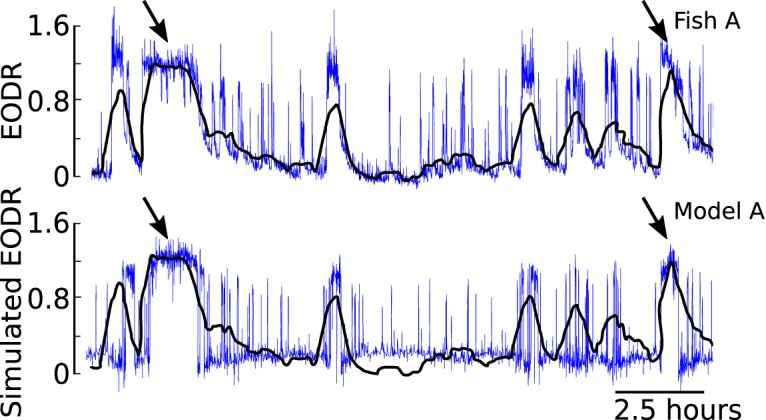
Simulation results from model A (lower panel, blue trace) qualitatively match the data from Fish A (upper panel, blue trace). This segment of data are taken from the trace in Figure 1. Applying a moving average filter to the observed EODR yields the black trace, which is then used as the latent variable, *s*(*t*), for model A (see Materials and Methods, Estimation of the latent variable). The black arrows are examples of active states that are expected to be reproduced by the model, since they are longer than the averaging window used to obtain the *s*(*t*).

**Figure 8.**
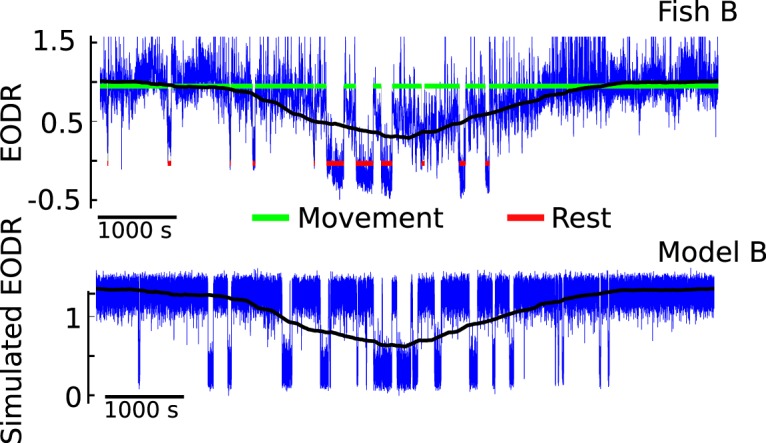
Simulation results from model B (lower panel, blue trace) qualitatively match the data from Fish B (upper panel, blue trace). Applying a moving average filter to the observed EODR yields the black trace, which is then used as the latent variable for model B (see Materials and Methods, Estimation of the latent variable).

To reproduce these results with our stochastic model, we first integrate Equation 1 for a duration similar to that of the pooled EODR time series, ∼60 h. In this case, the latent variable, *s*(*t*), is obtained by applying a moving average filter to this pooled time series (see Materials and Methods, Estimation of the latent variable). This method yields a realistic *s*(*t*) that evolves stochastically over a timescale much slower than that of the actual EODR (Figs. 7 and 8, black traces).

For Fish A, in addition to a time-dependent tilt parameter, *a*(*t*), we also assign a time dependency to the separation, *d*(*t*), both of which are linearly rescaled versions of the latent variable, *s*(*t*). This configuration creates a greater separation between the two stable points when the potential function is tilted toward the active state, and a smaller separation when it is tilted toward the inactive state. Note that, by construction, the separation, *d*(*t*), directly controls the transition amplitude. Also, since the level of tilt of the potential function strongly controls the durations of the active states (with, e.g., longer active states occurring when the potential function is tilted toward *x* = 1), simulations will show a positive correlation between the transition amplitude and the active state duration because *a*(*t*) and *d*(*t*) are both dependent on the same variable. For Fish B, on the other hand, only *a*(*t*) is dependent on *s*(*t*), with *d*(*t*) held constant.

These choices are based on the observations, described in the previous paragraph, that the transition amplitudes correlate with the active state durations for Fish A, but not for Fish B. It should be noted that, although the shape parameters, *d*(*t*) and *h*, of the (symmetric) double well can be held constant, the presence of a varying tilt parameter, *a*(*t*), inevitably causes fluctuations in the actual well depth and well separation of the potential function. We found, however, that these fluctuations are not by themselves able to explain the range of transition amplitudes observed in Fish A, which is why a time-dependent separation, *d*(*t*), was introduced for that case.

The time series obtained from these simulations qualitatively match those of the observed EODR (Figs. 7 and 8) and are processed in exactly the same way as the experimental data, i.e., they are fed through the state segregation scheme described in Materials and Methods, Data analysis. In the case of simulations, however, the EODR variance in the active state, *f*_4_, and the decay duration, *f*_5_, remain constant because variability of these features are not included in the model. Nevertheless, an analysis of these simulations yields scatter plots for both models that capture the correlation, or lack thereof, between the transition amplitude and the active state duration (Fig. 6*B*,*D*). By comparing model results and data in Figure 6, it is apparent that the observed transition amplitude has a greater variability than the simulated one. This is likely caused by the low number of parameters of the potential function used in our model, which makes it tighter than the “true” potential function. Adding parameters that could widen the bottom of the wells would allow for more variability in the simulated transition amplitudes.

**Figure 9.**
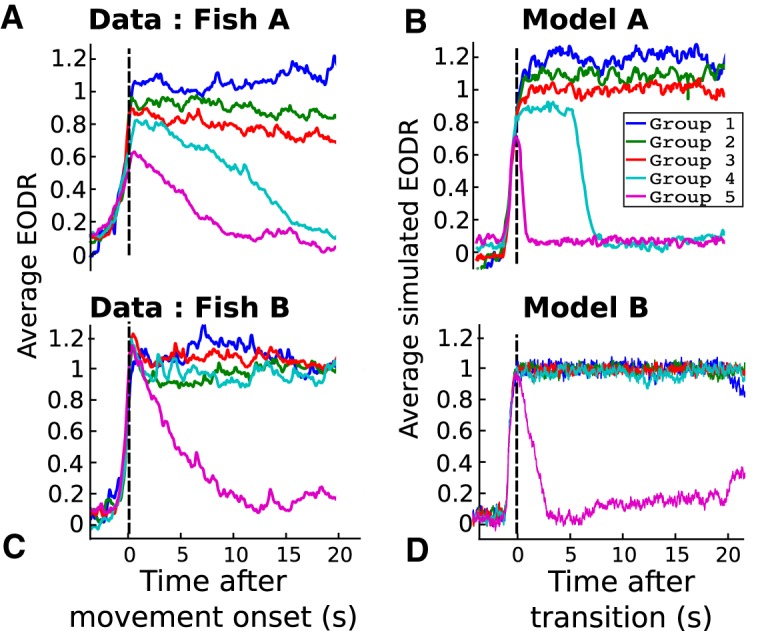
The models for both fish capture the essential features of inactive-to-active transitions across different groups. Coloured traces represent the average of a given group, i.e., the traces from panel *A* and *C* correspond exactly to the black traces of Figure 4. All active states are aligned with respect to movement onset time, in the case of experimental data, or with respect to the time of an upward crossing of the model’s unstable point, in the case of simulation (vertical dashed lines).

By using the segregation into groups described in Materials and Methods, Data analysis, we can plot the average traces for each group, observed and simulated (Fig. 9). We arrive at the same conclusion drawn from Figure 6, i.e., that Fish A transitions from inactive to active states with an amplitude that covaries with other attributes of the active states, as shown by the fact that different groups have different average transition amplitudes. In contrast, for Fish B, the transition amplitude is independent of the group. In addition, Figure 9 shows that the simulated time series exhibit a similar spectrum of active state shapes as the data.

From Figures. 4 and 9, one sees that some active states from the experimental data tend to follow a slow, almost linear decay toward the inactive state, which contrasts with the steeper upward transitions toward active states, see e.g., group 4 of Fish A in Figure 4. The analysis presented in this section does not attempt to reproduce this asymmetry, which is why the active states from the simulations show active-to-inactive transitions that are more abrupt than those of the data, as seen from the average traces of groups 4 and 5 (Fig. 9). See Discussion for potential additions to the model that could capture this asymmetry. In the next section, however, we show that a separate analysis, centered specifically around the movement offset times, offers a satisfying fit between simulated and observed active-to-inactive transitions.

### Offset-triggered analysis

We now examine in detail the structure of the EODR around movement offset times, that is, around active-to-inactive transition, for active states that belong to group 1, Fish A. This particular group of active states warrants special attention because it possesses attributes that convey unique information about the mechanisms that could govern these transitions.

**Figure 10.**
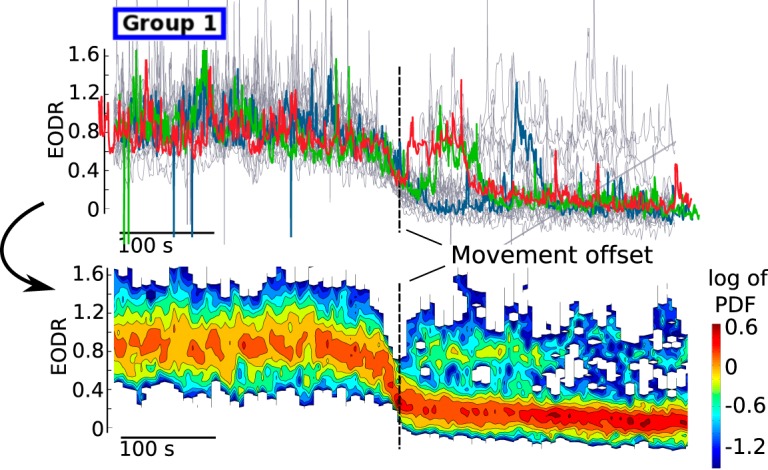
Group 1, from Fish A, shows a high propensity to return briefly to the active state shortly after the time of movement offset. This is superimposed on a slow, tail-like decay of the EODR. The gray traces in the upper panel correspond to the same active states that are shown in the upper left panel of Figure 4, but in this case, they are aligned with respect to the movement offset time, which allows a transition-triggered PDF to be compiled around movement offset (lower panel). Three representative traces in the upper panel are shown in color as examples.

For this particular group, we first wish to obtain the nonstationary probability density function (PDF) of the EODR, time locked to the time of movement offset, which we refer to as the transition-triggered PDF (see below, Transition-triggered PDF). This is obtained by first aligning each active state of this group with respect to the time of movement offset, and chopping off data that fall outside a 300-s time window centered around that time (Fig. 10, upper panel). The remaining set of EODR traces is then divided into several time bins, and within each bin we obtain a PDF for this segment of the EODR traces. Putting all these segments together yields a time-dependent PDF for the EODR, distributed around the time of movement offset (Fig. 10, lower panel). This analysis reveals two defining features of active-to-inactive transitions for this group of active states: (1) on average, the EODR undergoes a downward trend starting ∼1000 s before movement offset, and extending ∼1500 s beyond it (although in Figure 10, we only show ∼500 s around movement offset); (2) there is a significant probability that the fish briefly returns to an active state almost immediately after movement offset. These traits are absent from the other groups of active states and from other fish.

**Figure 11.**
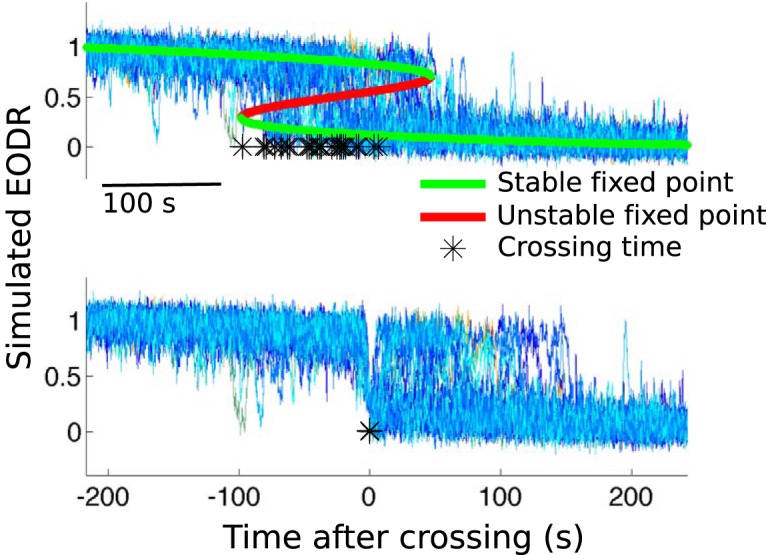
Top, Set of 30 Monte Carlo simulations of Equation 1, driven by a linear decay of the latent variable, *s_local_*(*t*). The stable and unstable point of the system are shown in green and red, respectively, and the black stars are time stamps marking downward crossings of the unstable point. The system is initialized at *x* = 1 and allowed to stabilize before the potential landscape starts to tilt over. All the realisations in the upper panel obey the PDF shown in Figure 12. Bottom, All traces from the upper panel are aligned with respect to the time stamps, which then allows the transition-triggered PDF of Figure 13 to be obtained.

To explain these results, we propose a dynamical scenario that involves a transient period of bistability caused by a slowly tilting double well potential. The associated deterministic dynamical system starts off with a single stable state that corresponds to the active state, then undergoes two saddle-node bifurcations that cause the appearance of the inactive stable state followed by the disappearance of the active stable state, and finally adopts its final configuration in the inactive state (Fig. 11, upper panel, red and green traces).

**Figure 12.**
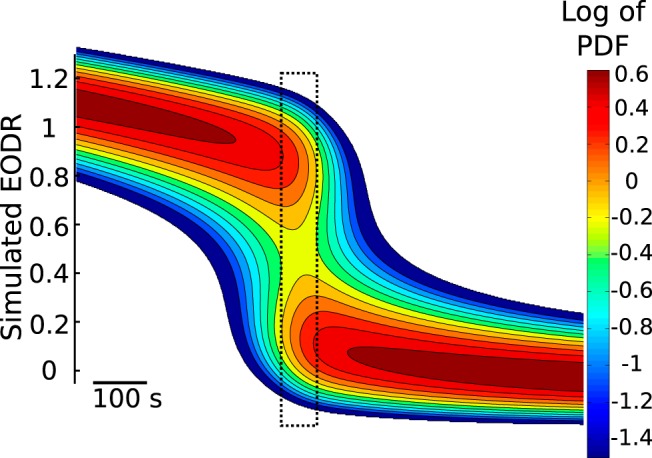
The nonstationary solution of the Fokker-Planck equation associated with Equation 1, driven by a linear decay of the latent variable, *s_local_*(*t*). The solution confirms the presence of a transient bistability period (dashed rectangle) that allows the system to briefly jump back to the active state immediately after a downward transition. The slope of *s_local_*(*t*), i.e., the tilting rate, controls the duration of this bistability period, with a higher rate leading to a briefer period. This solution is obtained numerically with a custom partial differential equation solver using finite volume discretization and implicit time-stepping (see Results, Numerical integration of the Fokker-Planck equation). Traces from the upper panel of Figure 10 evolve according to this PDF.

To show that this explanation reproduces the experimental results described above, we perform Monte Carlo simulations of Equation 1 under this scenario. In this case, because the timescale is so short, we simply prescribe a linear decay for the latent variable, *s_local_*(*t*), which yields a constant tilting rate for the double well potential function. This rate is varied until the simulations most closely match the experimental data (see Materials and Methods, Estimation of the latent variable). The system is initialized in the active state and forced by this progressive tilting. To complement the Monte Carlo approach, we also solve the Fokker-Planck equation associated with this nonstationary stochastic process and those initial conditions (see Results, Numerical integration of the Fokker-Planck equation). This solution confirms the presence of a brief, transient bistability period intercalated between two monostable regimes (Fig. 12).

For each realization of the nonstationary stochastic process, we assign a timestamp to the moment when the unstable fixed point of the system is crossed, and then align all the Monte Carlo realizations of the process with respect to this time (Fig. 11). The same procedure was applied to the experimental data in Figure 10, except that the movement offset time was used as the reference. Note that the realisations found in the upper panel of Figure 11 follow the nonstationary PDF of Figure 12. Once the realisations are aligned with respect to the transition times, however, they can no longer be compared with Figure 12, because they are now conditioned on a transition at time 0, rather than on the initial conditions used to solve the Fokker-Planck equation.

**Figure 13.**
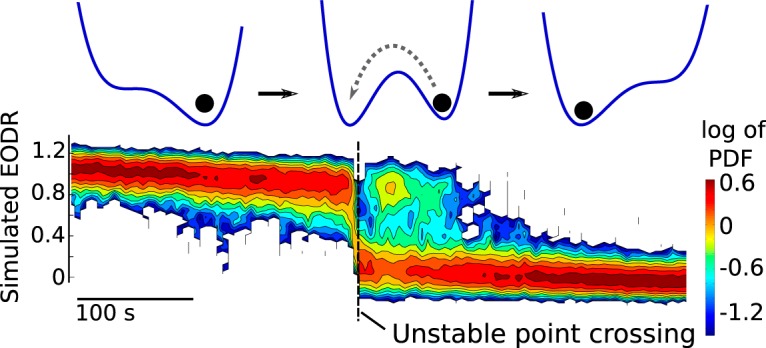
Monte Carlo simulations of the transiently bistable system yields a transition-triggered PDF that is qualitatively similar to that obtained from the data. The 30 iterations used to generate this distribution are first aligned with respect to the time when the unstable point of the system is crossed downward and are then processed in the same way as the experimental data to obtain the transition-triggered PDF shown here.

With the simulated EODR traces properly aligned, we now complete the analysis by obtaining the transition-triggered PDF for the simulated EODR (Fig. 13). This distribution is obtained in exactly the same way as the one shown in Figure 10 for the experimental data. The resulting comparison between Figure 13 (simulations) and Figure 10 (data) is satisfactory, with the model correctly capturing the slow downward trend of the EODR, as well as the tendency to return to the active state immediately after the transition. Note that by fine-tuning parameters associated with these Monte Carlo simulations, the match between data and model could be improved. Notably, by prescribing a nonlinear decay for *s_local_*(*t*), comprising, e.g., a plateau for the second half of the simulations, some bistability could remain for a longer time, allowing active-to-inactive transitions to occur >100 s following the transition time, similar to what is seen in Figure 10.

#### Transition-triggered PDF

With the active states from a given group aligned with respect to either the downward crossing of the unstable point (simulations), or the movement offset time (experimental data), we seek a PDF for the EODR, or simulated EODR, conditioned on a transition at a given time: for simulations, we seek p(x,t|′unstable point crossing at t = 0′), and for experimental data, p(x,t|′movement off set at t = 0′), where *x* is the simulated or observed EODR, t ∈ [−T/2,T/2], and where we choose *T* ∼ 500 s. To obtain this density, we bin each individual traces in 5 s windows, such that with our temporal resolution of 0.01 s and with 20 traces per group, we have access to 10,000 values per time bin. For each bin, we then obtain an estimate for the PDF by using Matlab’s kernel density estimator (“ksdensity” function, default options). Putting all time bins together, we can show the evolution of the PDF with a colorplot, centered on the desired transition time.

#### Numerical integration of the Fokker-Planck equation

The Fokker-Planck equation associated with Equation 1 is

(4) $$\frac{\partial p(x,t)}{\partial t}=\frac{\partial }{\partial x}\left[\frac{\partial U(x,t)}{\partial x}p(x,t)\right]+D\frac{\partial^{2}p(x,t)}{\partial x^{2}}$$

where *p*(*x*, *t*) is the PDF of *x*(*t*) given the initial condition *x*(*t* = 0) = 1, and where the nonstationarity embedded in *U*(*x*, *t*) is conveyed by the latent variable, *s*(*t*). We obtain the nonstationary solution of this equation by numerical integration with a custom partial differential equation solver that implements a finite volume discretization with the fully implicit Euler scheme. The advective term is treated with the upwind scheme and a linear interpolation profile for the spatial derivative of *p*(*x*, *t*) is applied to the diffusive term (Patankar, 1980). The resulting algebraic equation is solved with the tridiagonal matrix algorithm (Patankar, 1980). The spatial resolution Δ*x* is 0.004, for a total of 1000 grid points between *x* = −2 and *x* = 2. The time step is the same as for the integration of Equation 1, 0.01 s. Note that, although the solution of this equation (Fig. 12) looks similar to the transition-triggered PDF obtained from the simulations (Fig. 13), they should not be compared together. Although they are both nonstationary PDFs, they are not conditioned on the same event: the former is conditioned on *x*(*t* = 0) = 1, with t ∈ [0,T], while the latter is conditioned on a transition occurring at *t* = 0, with t ∈ [−T/2,T/2].

### RTDs

For this final analysis, we focus on the RTDs, that is, the PDF for the time spent in a given state. We show that our proposed modeling framework produces RTDs that are consistent with those of the data. Here, we focus primarily on the inactive state RTDs. This is because fish movement during the active state might cause re-afferent signals that would interfere with the neural process governing the termination of movement. Notably, the possibility of the fish encountering the tank walls is an unavoidable element that might invalidate our assumption that an open-loop subsystem is responsible for terminating movement (see assumption 1). During the inactive state, however, the fish’s sensing volume remains unchanged and our working assumptions hold. Unless specified otherwise, the inactive state is thus implicitly assumed for the remainder of this section.

Recall that the experimental data from each fish is divided into several recording sessions. Comparing the RTDs obtained from each of these sessions with the RTD of the pooled time series reveals significant statistical differences between them. This prevents us from assuming that the residence times from different recording sessions are sampled from the same underlying PDF. We thus refrain from pooling those residence times and rather opt for analyzing each recording session separately.

For all fish, we find that the stretched exponential family of PDFs provide a satisfying fit for the RTDs (Table 2, second column). Stretched exponential distributions lie on a continuum between exponential and power-law distributions, with a single parameter controlling which regime is more expressed (see below, Stretched exponential fitting). Except for the limiting exponential case, they possess a long tail and are often used to describe scale-free phenomena (Luevano, 2013).

**Table 2:** Comparison between the fitting statistics for the inactive state RTD**s of the data and of model results, for each recording session**

Data					Simulations			
Fish-session	*p* value	Numebr of states	α	<t> (s)	*p* value	Number of states	α	<t> (s)
A-1	0.27	49	0.30	473.14	0.31 ± 0.34	43.74 ± 6.23	0.41±0.34	473.21 ± 81.43
A-2	0.37	30	0.27	523.30	0.31 ± 0.32	32.34 ± 4.49	0.33 ± 0.22	466.15 ± 77.94
A-3	0.03	51	0.24	773.87	0.25 ± 0.31	68.21 ± 7.47	0.34 ± 0.20	508.46 ± 64.71
A-4	0.59	93	0.29	529.59	0.20 ± 0.28	80.59 ± 8.66	0.38 ± 0.21	542.10 ± 75.30
A-5	0.45	67	0.47	609.23	0.24 ± 0.30	67.76 ± 7.01	0.38 ± 0.27	542.22 ± 66.51
A-6	0.67	31	0.52	524.04	0.27 ± 0.31	36.82 ± 5.24	0.44 ± 0.37	414.97 ± 68.59
A-7	0.71	72	0.42	439.88	0.17 ± 0.25	72.38 ± 8.14	0.32 ± 0.20	408.15 ± 60.43
B-1	0.65	180	1.00	32.18	0.47 ± 0.26	73.70 ± 6.62	0.56 ± 0.17	53.33 ± 7.42
B-2	0.77	99	0.93	25.05	0.44 ± 0.25	55.30 ± 7.72	0.62 ± 0.24	42.12 ± 7.21
B-3	0.51	81	0.77	34.65	0.54 ± 0.26	71.53 ± 6.52	0.71 ± 0.22	40.26 ± 5.23
B-4	0.78	109	0.61	34.36	0.49 ± 0.25	56.20 ± 6.22	0.57 ± 0.19	51.83 ± 8.30
B-5	0.37	35	0.55	34.55	0.44 ± 0.25	36.89 ± 5.89	0.58 ± 0.30	40.94 ± 9.41
B-6	0.91	72	0.84	77.18	0.49 ± 0.25	76.27 ± 7.75	0.57 ± 0.15	73.89 ± 10.15
B-7	0.69	77	0.45	50.97	0.45 ± 0.24	70.58 ± 7.70	0.54 ± 0.16	58.93 ± 8.74
B-8	0.75	34	0.33	71.89	0.47 ± 0.27	61.93 ± 7.36	0.55 ± 0.18	63.61 ± 10.50

The *p* values are from two sample Kolmogorov-Smirnov tests between the empirical RTD and the best fit stretched exponential distribution. *α* and < *t* > are parameters of the stretched exponential distribution of Equation 6. Simulation results are formatted as mean ± SD, as obtained from 100 iterations of Equation 1, driven by the *s*(*t*) associated with each recording session.

To determine whether our modeling framework also fits RTDs with stretched exponential distributions, we use the same approach as in the Onset-triggered Analysis, where the latent variable *s*(*t*) is given by the smoothed EODR (Figs. 7 and 8, black traces). The only difference in this case is that we obtain several traces for *s*(*t*), one for each recording sessions, rather than extracting a single trace from the pooled EODR time series. Each *s*(*t*) trace is then used to drive Equation 1 and thus to obtain a simulated EODR associated with a given recording session. These simulation results are then piped through the same transition detection algorithm as the data, from which we extract the residence times and compile the RTDs for each recording session. The resulting RTDs are then fitted to stretched exponential distributions by the same procedure applied to the data. To obtain proper statistics on the simulated RTDs, we repeat this procedure to obtain 100 iterations of the simulated EODR. From this ensemble of simulation results, we extract, for each recording session, the average *p* value evaluating the stretched exponential fitting, the average number of states, and the average parameters of the fitting distribution. We report these average values in Table 2, and compare them to those of the experimental data associated with the same recording session.

Not only are the simulated RTDs well-fitted by stretched exponentials like the data, but they also closely resemble the RTDs of their associated recording sessions, regardless of which fish is considered, as shown in Figure 14*A* for two examples of recording sessions. As mentioned in Materials and Methods, Derivation of the nonlinear stochastic model, however, the fit between the simulated and the data RTDs for large timescales should not be interpreted as model validation, since we have used a large averaging time window to infer *s*(*t*) from the data. The length of this time window is shown as the black dotted vertical line in Figure 14. From the ensemble of 100 simulation results associated with each recording session, we can also obtain PDFs for the fitting parameters, *α* and < *t* >, which are then compared with the observed values of these parameters, confirming the correspondence between experimental data and model results (Figure 14*B*). In most cases (21 parameters out of 30), the observed parameter values fall within 1 SD of the simulated ensemble average (Table 2). The match between observed and simulated RTDs also holds for the active state, but in this case about half of the recording sessions are not well-fitted by stretched exponentials, although they still possess a long tail.

**Figure:**
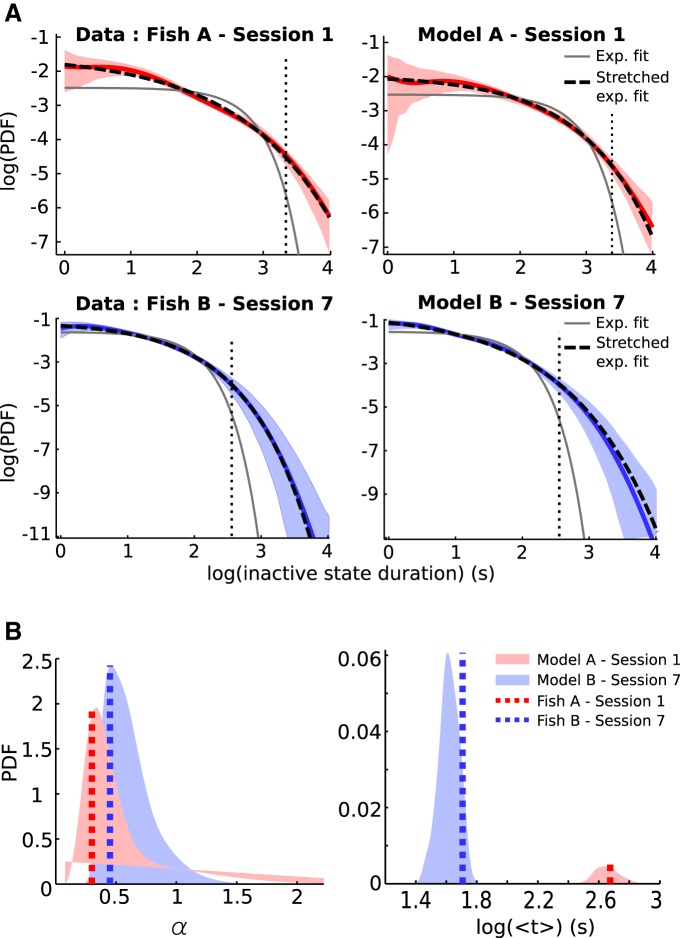
Figure 14. ***A***, Inactive state RTDs. Models for both fish produce inactive state RTDs (coloured curves) that are consistent with those of the data, all of which are fitted by the stretched exponential distribution functions of Equation 6 (dashed curves). For comparison, gray curves show the best fit exponential densities for the RTDs. Fitting parameters for all sessions can be found in Table 2, for both experimental data and simulations. Shaded areas are 95% bootstrap confidence interval for the RTDs, obtained with Matlab’s “bootci” function (1000 bootstrap samples). The simulated RTDs shown here are obtained from a single realisation of Equation 1, driven by the *s*(*t*) associated with the appropriate recording session. The dotted vertical lines correspond to the value of the averaging window used to obtain *s*(*t*). ***B***, Distribution of fitting parameters. These distributions are obtained from the ensemble of 100 iterations associated with the same sessions as in ***A***. Dashed lines show the value of these parameters obtained from fitting the experimentally observed RTDs. These values correspond to the fourth and fifth columns of Table 2.

Lastly, as the appearance of stretched exponential distributions can be associated with long-range correlations (Luevano, 2013), we examine whether there exist correlations between the duration of successive inactive states (i.e., duration of the *i^th^* inactive state against that of the (*i* – 1)*^th^* one). For Fish A, we find no such correlations, in neither model or data. For Fish B, however, a small but significant correlation is observed (data: *r* = 0.13, *p* = 6.69 × 10^– 4^; model: *r* = 0.23, *p* = 1.89 × 10^–15^). For both data and model these correlations persist up to a lag of six to eight inactive states. This shows that, for Fish B, the latent variable *s*(*t*) imparts some degree of memory on a slow time scale to the faster dynamics of the EODR. The fact that these correlations are absent for Fish A can be explained by the lower level of noise, *D*, for this individual (see above, Integration of the stochastic differential equation). Indeed, less noise means that less transitions are triggered, which in this case means that the EODR has less opportunities to “sample” and act as a readout for the latent variable. This is in line with the notion of aperiodic stochastic resonance (Collins et al., 1996), where there is an optimal level of noise at which the fast variable of slowly and aperiodically driven bistable system is an optimal readout of the driving force.

#### Stretched exponential fitting

The stretched exponential distribution is given by:

(5) $$p(t)=K\exp(-\beta t^{\alpha })$$

Following Luevano (2013), the prefactor *K* and constant *β* can be expressed in terms of the exponent *α* and the mean value < *t* >:

(6) $$p(t)=\frac{\alpha b}{\Gamma (1/\alpha )<t>}\exp(-{\left(bt/<t>\right)}^{\alpha })$$

where $b=\frac{\Gamma (2/\alpha )}{\Gamma (1/\alpha )}$. With knowledge of < *t* >, the stretched exponential is thus characterized by a single parameter, *α*. This property is particularly useful for fitting purposes, since we have access to an estimate of < *t* >. Given a set of residence times, from either the data or simulations, its empirical PDF, $\tilde{p}(t)$, is estimated via kernel density estimation (ksdensity function in Matlab, ’support’ option set to ’positive’) and the associated mean is calculated as $<t>=\int_{0}^{\infty }t\tilde{p}(t)dt$. A 1-D grid search along possible values of *α* is then performed to minimize the squared difference between the estimated density $\tilde{p}(t)$ and the candidate distribution of Equation 6. The quality of the fit is evaluated with a two sample Kolmogorov-Smirnov test (“kstest2” function in Matlab, default options), whose *p* values are reported in Table 2.

## Discussion

We have shown that a low-dimensional modeling framework, consisting of only a small set of dynamical components, captures the core aspects of spontaneous transitions between active and inactive behavioral states in electric fish. Given that the Jun et al. (2014) dataset shows signs of nonstationarity, we developed a scheme that segregates similar segments of data together, allowing us to properly investigate the mechanism from which stereotyped shapes of active states emerge. We propose that this data analysis scheme is a useful tool to break down and understand nonstationarity in stochastic data. By applying this scheme, we examined in detail the average structure of the EODR around times of movement onset and offset.

For transitions around movement onset, our analysis revealed a positive correlation between the transition amplitude and the duration of the active states (for Fish A). By introducing a time dependency for both the tilt and separation variable of the potential function, our model correctly captures the observed correlation. In the case of transitions around movement offset, our data analysis scheme allowed us to observe the presence of a brief period, immediately following movement offset, where fish show a propensity to return to the active state. To explain this, we proposed a simple dynamical scenario where the potential function is tilted toward the inactive state with a constant rate. This creates a brief period of bistability that allows the simulated EODR to briefly return to the active state following an active-to-inactive transition, as seen in the data. Finally, we showed how simulating the model over long time scale yields time series where the RTDs are long tailed and well fitted by stretched exponential distributions, and where correlations emerge between the duration of succesive states (for Fish B only). Both these modeling results are consistent with the data.

Together, these analyses lead us to conclude that low-dimensional, bistable neural dynamics underly the emergence of spontaneous transitions between behavioral active and inactive states in electric fish, and that stochastic threshold crossing is the mechanism triggering these transitions. These findings corroborate recent results that also confirm a key role for fluctuating neural activity in the timing of walk/rest transitions in freely walking *Drosophila* (Maesani et al., 2015). In addition, a major conclusion of our work is the identification of a nonstationary latent variable that exerts its influence through the shape of the bistable potential landscape governing the transition dynamics. We hypothesize that this bistability is established in highly recurrent telencephalic networks, and that neuromodulation deriving from diencephalic peptidergic systems provides the inferred nonstationary forcing to this network (Trinh et al., 2016; Elliott et al., 2017).

### Putative neural structures involved in the transitions

Lesions of the telencephalon leave electric fish in the inactive state (Pereira et al., 2014), suggesting that it contains the circuitry responsible for the stochastic switching between inactive and active states. In support of this hypothesis, a recent study has shown that the recurrent networks of the gymnotiform telencephalon can be induced to switch between up and down states (Elliott and Maler, 2015). Furthermore, work in zebrafish suggests that the orexin and galanin peptidergic systems of the hypothalamus can control the probability of such switches (Prober et al., 2006; Woods et al., 2014). The slow fluctuations of the latent variable that modulates the relative depth of the wells in our model may therefore be caused by these peptidergic neurons. Orexin (goldfish, zebrafish; Kaslin et al., 2004; Huesa et al., 2005) and galanin (gymnotiform; Yamamoto and Maler, 1992) neurons are located in the lateral hypothalamus and project to a region of the ventral telencephalon homologous to the mammalian basal ganglia (Harvey-Girard et al., 2013). The basal ganglia appears to be the core telencephalic region essential for initiating specific motor output (Grillner et al., 2005) and, in both mammals (Grillner et al., 2005) and gymnotiform (Giassi et al., 2012b; Trinh et al., 2016; Elliott et al., 2017), the basal ganglia receives input from dorsal telencephalic recurrent excitatory neural networks. We therefore hypothesize that (1) the dorsal telencephalon contains the recurrent networks responsible for the stochastic switching and these networks drive the ventral telencephalon; (2) movement is initiated by the ventral telencephalon; (3) the activity of the ventral telencephalon is modulated, on a slow time scale, by input from the orexin and galanin neurons of the lateral hypothalamus. This may not be an entirely open loop system because the lateral hypothalamus of gymnotiform itself receives a strong input from dorsal telencephalon (Giassi et al., 2012a). Further work into the details of the entire network will, however, be required to elucidate how such descending input might regulate behavioral state transitions.

### Limitations of the model

As our goal is to capture the core phenomenology of the Jun et al. (2014) data with a minimal set of assumptions, some features of the data inevitably fall outside the scope of our modeling framework. Notably, we do not attempt to reproduce the increased EODR fluctuations in the active state (Fig. 2), nor the variable intensity of these fluctuations, as quantified by *f*_4_ (Fig. 3*A*). Although implementing a mapping between the latent variable and the width of the active state well could resolve this discrepancy, we deem the available data as insufficient to rigorously constrain such an ad hoc addition to the model. A biophysically motivated description of the neural dynamics would be required to properly address inquiries into these finer details of the data. To our knowledge, no such modeling efforts have been made in the context of intermittent locomotion. However, given the apparent two-state and stochastic nature of the EODR, we can draw parallels with the phenomena of cortical up and down states, which also show higher levels of fluctuations in the up state (Wilson and Kawaguchi, 1996).

Models of up-down state transitions typically comprise an excitatory and a regulatory component (e.g., inhibition, short term depression, or adaptation), also known as activator/repressor dynamics (Hidalgo et al., 2012). The interplay between both components establishes bistability in the network activity and allows stochastic switching between stable states once noise is added to the system (Holcman and Tsodyks, 2006; Mejias et al., 2010). Although these up-down state transitions occur at around 0.5-2 Hz, much shorter durations than those of the behavioral state transitions examined in our article, similar dynamical principles might be able to explain both phenomena. For instance, to explain the enhanced fluctuations in up states, Hidalgo et al. (2012) suggest a general mechanism that entails stochastic perturbations of the system around the up state, for which the associated fixed point is a stable focus (Hidalgo et al., 2012). This results in the amplification of a resonant frequency that is absent from the down state, where the fixed point is rather a stable node. The idea to represent the up state as a stable focus has also been developed for a purely deterministic system (Ghorbani et al., 2012). These studies suggest that adding a repressor variable to our model, in such a way as to have the active state fixed point become a focus (which requires two-dimensional dynamics locally), might be sufficient to capture this aspect of the Jun et al. (2014) data.

Adding a second dimension to the model might also help to capture the asymmetry observed in the shape of individual active states: most of them show a slow downward trend long before the movement offset time, followed by an active-to-inactive transition period that is less abrupt than that of the inactive-to-active transitions (Figs. 1, insets, and 4). We also speculate that, in a 2-D context, differences in timescales for the activator and repressor variable might cause the path toward the active state to be different from the one leaving it. Although a two-variable system might be needed to achieve this asymmetry, we believe that the 1-D analyses conducted in this article remain valid in the sense that trajectories to and from the active state might be governed by distinct 1-D dynamics local to each branch (see Pikovsky and Kurths (1997) for an example of how a 1-D description can approximate part of a stochastic 2-D trajectory). This is why we analyzed transitions around movement offset independently of those around movement onset.

### Spontaneous movement as decisions making

Because movement initiation fundamentally emerges from decision-making processes (Shadlen and Kiani, 2013), quantitative models of decision-making might provide an adequate substrate to understand the randomness observed in intermittency. So-called accumulator models, for instance, have been extensively and successfully applied to perceptual decision-making tasks (Wang, 2008). Yet, their validity with respect to spontaneous movements has only been suggested recently in Schurger et al. (2012). The authors of that study contend that the well-known readiness potential (Kornhuber and Deecke, 1965; Libet et al., 1983) that precedes the initiation of movement reflects ongoing spontaneous fluctuations in neural activity, rather than activity related to motor preparation and planning, as traditionally believed. To support this claim, they show that a 1-D model, the leaky stochastic accumulator, with a threshold applied to its output, provides a satisfying fit to EEG data from spontaneous movement tasks. In formal terms, their model is a biased Ornstein-Uhlenbeck process, which describes the evolution of a Brownian variable in a parabolic potential landscape. Although our model variable evolves in a double well potential landscape, the trajectory near either attractor states does approximate an Ornstein-Uhlenbeck process. With the unstable point of the double well acting as a threshold, the model we present here is therefore in the same spirit as that of Schurger et al. (2012). We thus believe that our contribution supports their conclusions, and that spontaneous movement initiation in electric fish is also governed by ongoing fluctuations, with threshold crossing as the mechanism triggering the initiation of movement.

### Long tail RTDs in intermittent behavior

A large variety of spontaneous animal behavior, including intermittent locomotion, is known to be scale-free with power-laws describing the duration distributions of certain behaviors (Harnos et al., 2000; Mashanova et al., 2010; Bazazi et al., 2012). It has been shown in Proekt et al. (2012) that this is a generic trait of a broad class of nonequilibrium Markovian systems. According to this study, the essential requirement is the existence of a macroscopic timescale that imposes an upper bound on the duration of behavioral states and beyond which scale invariance breaks down, which they suggest might arise from species-specific metabolic processes. They noted that the ubiquity of scale invariance in spontaneous behavior suggests the existence of “an elementary un-differentiated process in the nervous system that governs activation of all behaviors [sic].” We believe the model presented herein provides such a general mechanism: by continuously modulating the switching rates between attractor states, the nonstationary latent variable, interacting with neural noise, is read out as a behavioral driver that evolves over a wide range of timescales, giving rise to the long tail nature of the RTDs. We speculate that the slower of these timescales, that of the latent variable itself, fulfills the role of the macroscopic time scale suggested in Proekt et al. (2012).

In the case of electric fish, the RTDs are stretched exponentials, but our modeling framework could also generate power-law RTDs. Indeed, it has been shown that a stochastic bistable system with a nonstationary energy barrier, similar to the model we present here, can yield power-law RTDs in certain regimes (Tu and Grinstein, 2005). This emergence of power-law and stretched exponential distributions in nonstationary bistable systems can be understood in light of the fact that (1) continuously modulating the energy barrier introduces a distribution of switching rates over the course of the experiments, (2) individually, these switching rates translate into exponentially distributed residence times (Gammaitoni et al., 1998), and (3) it has been shown that sums of exponentials can approximate long tail distributions (Feldmann and Whitt, 1998; Liebovitch and Tóth, 1991; Johnston, 2006). In addition, an approach similar to ours has been effective in explaining the appearance of power-law and long-tailed RTDs in transitions between cortical up and down states (Mejias et al., 2010).

### Future work

As *in vivo* recordings of telancephalic activity during spontaneous behavior become available, it should be possible to constrain a biophysically motivated neural network model to describe the action selection circuitry subserving movement initiation and termination. This could help elucidate the precise mechanism by which bistable attractor dynamics is established in the network, thereby exposing a correspondence between biophysical parameters and the potential function introduced in this article.

Genetic manipulation of orexin and galanin neurons of the zebrafish hypothalamus may also directly test whether these peptides provide the slow modulatory variable that controls state switching. This could yield a biophysical model of the slow latent variable, which would be used as input to the bistable network. This could help understand the neurophysiological basis of how neuromodulation affects the potential function.

Furthermore, the analyses presented here could be refined by considering a richer behavioral space than the binary, active-inactive classification that we used here. With continuous video tracking, other behavioral states can be used to quantify fish behavior, such as backward swimming and turning. Based on the transition probability matrix obtained from this description, it might be possible to propose a stochastic multistable attractor model to describe more fully the observed spontaneous behavior.

Finally, although we have exclusively focused on spontaneous behavior, it remains to be understood how intrinsic behavioral drivers interact with sensory inputs, i.e., what sort of mechanism implements the influence of sensory perturbations? Is it mediated only through neuromodulation, as suggested by the induced up states of the telencephalon (Elliott and Maler, 2015), or is there a more direct pathway interacting with the inferred attractor dynamics? In other words, one might want to eventually include closed loop features, going beyond the open-loop perspective that underlies the work presented here, to gain a better understanding of how behavioral state transitions occur in more complex settings. Our work also provides an adequate framework to understand sensory responsiveness: depending on the configuration of the potential function and the height of the energy barrier, fish might be more or less inclined to undergo a behavioral state transition following sensory input.

## Synthesis

Reviewing Editor: Elizabeth Cropper, Mt Sinai Sch Med Decisions are customarily a result of the Reviewing Editor and the peer reviewers coming together and discussing their recommendations until a consensus is reached. When revisions are invited, a fact-based synthesis statement explaining their decision and outlining what is needed to prepare a revision will be listed below. The following reviewer(s) agreed to reveal their identity: Björn Brembs, Jan Benda.

As you will see in more detail below Reviewer #1 was very enthusiastic about this work but notes that he is not competent to comment on the technical parts of the model. He makes a “conceptual” suggestion that might help non-modeling readers understand the work. Please address this either by revising the manuscript or commenting on it in the response letter.

In contrast, Reviewer #2 thought the idea behind the manuscript was interesting but that the work was incomplete. Consequently, we sent the manuscript to a third reviewer. As is indicated in more detail below the third reviewer agreed with Reviewer #1. The bottom line is that we are not asking for additional statistics. Reviewer #3 does however ask that you discuss a specific issue. To quote “However, there might be a problem with respect to predicting the long tails of the residence times. If the tails are longer than the timewindow used for computing the latent variable, the model does no longer predict the residence times - they are given by the data. This should be carefully discussed.” Please address this comment. As you will see below all other points raised were minor.

Reviewer #1

I have reviewed this work in a previous version. This submission is substantially revised and amended, also incorporating most of my previous comments. I already liked the first version, this one is even better.

This is a very timely work on data from a wonderful experiment. These kinds of study are important as they serve to help crack one of the most fundamental nuts in neuroscience: how do brains generate output without sensory input? I find the approach very reasonable - it is in principle analogous, albeit technically much more sophisticated, to our approach in Maye et al. (cited by the authors). From their description, I cannot find a flaw in their approach. On the contrary, it looks creative, meticulous and very thought-through. I thoroughly enjoyed reading the manuscript - their model is beautiful with great intuitive explanatory power.

As Alexander Maye did all the computational work for our paper, I'm not competent to comment on the technical merits of the model. I will therefore comment from the perspective of a behavioral neuroscientist. I like this paper a lot and have only one conceptual suggestion for the authors, namely to help non-modeling readers to understand and appreciate their work.

The ability to distinguish random time series from nonlinear time-series has been a key development of nonlinear forecasting methods in the 1990s, see, e.g., doi:10.1038/358217a0 or doi:10.1038/344734a0. In the light of these efforts, distinguishing between randomness and stochasticity is vital for further exploitation of the underlying neurobiological mechanisms of behavior. If this distinction is not made, the value of the model in terms of understanding spontaneous behavior will be severely limited. Granted, the play down the value of their own model by writing (l327): “There is rather a preliminary need to characterize the key dynamical features of the data from a phenomenological perspective.” However, I think it makes a significant difference for the kind of future experiments being made, if our models do not discern between stochastic processes and nonlinear processes, as both would be implemented radically differently in biology. Having read the entire article, I have no doubt that the authors skillfully and conscientiously combined random and nonlinear processes in their model. However, at least for me as an experimental neuroscientist, this combination is too implicit and only becomes clear after reading the entire manuscript. I tend to think that most of my experimental colleagues would benefit from a much more explicit, verbal distinction (starting in the title!) between a) random or stochastic processes, b) nonlinear/deterministic processes and c) processes where there are both components combined and in what way they were combined.

To exemplify my point, may I explain the model's impact on my understanding? A central conceptual aspect of the work lies in how the model presented here is perceived by experimentalists. We (Maye et al.) also found several stochastic processes to be able to replicate certain simple features of spontaneous, in our case fly turning, behavior, similar to the way the authors' model captures the fishes' Residence Time Distributions. Indeed, there are many different models in the literature that capture the heavy-tailed (stretched exponential) 1st order statistics so often found in animal behavior. Inasmuch as I understand the model, it incorporates a nonlinear shape function that modulates the outcomes of the stochastic base of the model. Or, conversely, a stochastic process operating upon the constraints of an essentially nonlinear core. In that regard it appears related to some of the models we have tested. However, their model is obviously much more refined, using more detailed and specific assumptions. Given I'm not a modeler, I may have misunderstood the technical part of the paper. I wonder why the authors chose to call it a stochastic model? I may have also misunderstood the model description, but couldn't the model have just as well been called a noisy nonlinear model? In fact they use nonlinear nomenclature (e.g. attractor dynamics, etc.) to describe the assumptions and workings of their model. As I understand it, the bistable potential function is a simplification to avoid complex recurrent network models. It is thus there to model the nonlinear network dynamics, without directly invoking recurrent network models. As such, can't one just see the stochastic component as just a small grain of noise added to an essentially nonlinear model? The authors seem to hint that the addition of noise is what makes such models behave in a more random-like fashion (l841-844): “The interplay between both components establishes bistability in the network activity and allows stochastic switching between stable states once noise is added to the system (Holcman and Tsodyks, 2006; Mejias et al., 2010).” Given the cooperation between nonlinear and stochastic processes in the model, the decision is a little bit akin to the one between a glass half full and one half empty. However, in this case, nomenclature influences how we think about a problem and how we think about a problem influences what experiments and models we will pursue next. Hence, the decision of how to call their model may be influencing the coming flow of both experimental and theoretical research in this area.

Along similar veins, shouldn't the possibility of overestimating the contribution of noise to the overall behavioral state of the animal by discretizing essentially continuous processes at least be mentioned?

Minor:

Fig. 3b: What about the black dots?

Fig. 4: I fear readers like me might need a better explanation of the grouping, as groups look very different between fish A and B. E.g., in Fish A, each group looks different, while all of them show a ramp up before movement onset. In Fish B, group 1-4 look indistinguishable with ramps largely after movement onset. Only group five looks remotely similar between fish A and B. If I were presented with the groups for fish B alone, I'd lump groups 1-4 in the same group by visual inspection and would note that the EODR ramp always occurs after movement onset - with ensuing consequences for cause-and-effect hypotheses. IOW, Fish B only shows two groups. BTW, Fig. 9 does not seem to reflect the difference in ramp wrt movement onset?

Fig. 7: Why is the movement/rest indication not shown in all panels?

It seems the smoothing algorithm (low pass) the authors are using may change the slope of the data trace? Fig. 7 seems to show a nice example of that. I what way may this change the dynamics of movement on/offset?

The authors state on l575/576: “i) on average, the EODR undergoes a downward trend starting ~1000s before movement offset, and extending ~1500s beyond it” However, these time ranges are outside the data shown in Fig. 10.

Reviewer #2

Although the idea behind this manuscript is interesting and could imply an important advance in the field, I think the present work is incomplete and the reported results seem to be trivial with make it unsuitable for publication in a high impact journal as it is Eneuro.

More precisely, the theoretical model introduced to describe transitions between active and inactive behavioural states is incomplete since the main variable to describe te non-stationarity of these transitions and their dynamical features, namely s(t), is taken as the moving average filtered version of the EODR experimental time series. In this way, the model works as a slave of the experimental time series. Therefore, is not surprising that its outcome will well reproduce these experimental time series since experimental dynamics is included in the model through s(t).

It would have been more interesting to cosider a pure theoretical model dynamics for s(t) coupled with dynamics (1), perhaps biophysically motivated or including non-linearities determined with a deep analysis of the dynamical features of the experimental time-series.

Reviewer #3

The manuscript is a very detailed analysis of a great dataset on spontaneous transitions of behavioral states. The procedures are very clearly explained and it is fun to read.

I completely agree with reviewer #1.

With regard tow reviewer #2: yes, the slow variable is taken from the data. But the key idea of the model is the fast timescale, namely the transition of the behavioral states in the double well - as it is stated in the title. The double well is modified by the slow variable. On a shorter time scale the double well model nicely reproduces the switches, including the many switches that are not captured at all by the slow variable.

The manuscript is already long, therefore I would NOT suggest to add some statistics of the slow process - that could be a different manuscript.

However, there might be a problem with respect to predicting the long tails of the residence times. If the tails are longer than the timewindow used for computing the latent variable, the model does no longer predict the residence times - they are given by the data. This should be carefully discussed. Also in Figure 14 the duration of the averaging window should be marked.

Minor issues:

line 123: “useful to consifer EODR” -> “useful to consider EOD rate (EODR)”

line235: you normalized EODR data to show transitions between 0 and 1. Still you have the mean rate of the active state f3 as a parameter. Can you briefly comment on why this is not normalized out.

line 242: can you show how PCA1 depends on the parameters f1-f5?

Figure 4: by looking on fish A it looks like the initial slope of the up state might strongly correlate with the duration of the up state. That would imply that already at onset of the transition the offset is kind of pre-determined. You may comment on this.

My impression is that the details dimensionality reduction scheme is not so important for the model but fig 2 and 4 are important). This section might be shortened. THis is just a suggestion, I am fine either way.

## References

[B1] Bazazi S, Bartumeus F, Hale J, Couzin I (2012) Intermittent motion in desert locusts: behavioural complexity in simple environments. PLoS Comput Biol 8:e1002498. 10.1371/journal.pcbi.1002498 22589707 PMC3349720

[B2] Berman GJ, Choi DM, Bialek W, Shaevitz JW (2014) Mapping the stereotyped behaviour of freely moving fruit flies. J R Soc Interface 11:20140672. 25142523 10.1098/rsif.2014.0672 PMC4233753

[B3] Bullock TH, Hopkins CD, Popper AN, Fay RR (eds) (2005). Electroreception. Springer Handbook of Auditory Research. Vol 21. New York: Springer.

[B4] Caputi AA, Aguilera PA, Castelló ME (2003) Probability and amplitude of novelty responses as a function of the change in contrast of the reafferent image in G carapo. J Exp Biol 206:999–1010. 10.1242/jeb.00199 12582142

[B5] Collins JJ, Chow CC, Capela AC, Imhoff TT (1996) Aperiodic stochastic resonance. Phys Rev E 54:5575–5584. 9965744 10.1103/physreve.54.5575

[B6] Curto C, Sakata S, Marguet S, Itskov V, Harris KD (2009) A simple model of cortical dynamics explains variability and state dependence of sensory responses in urethane-anesthetized auditory cortex. J Neurosci 29:10600–10612. 10.1523/JNEUROSCI.2053-09.2009 19710313 PMC2861166

[B7] Deco G, Martí D, Ledberg A, Reig R, Sanchez Vives MV (2009) Effective reduced diffusion-models: a data driven approach to the analysis of neuronal dynamics. PLoS Comput Biol 5:e1000587 10.1371/journal.pcbi.1000587 19997490 PMC2778141

[B8] Elliott SB, Harvey-Girard E, Giassi ACC, Maler L (2017) Hippocampal-like circuitry in the pallium of an electric fish: possible substrates for recursive pattern separation and completion. J Comp Neur 525:8–46. 10.1002/cne.24060 27292574

[B9] Elliott SB, Maler L (2015) Stimulus induced up states in the dorsal pallium of a weakly electric fish. J Neurophysiol 114:2071–2076. 10.1152/jn.00666.2015 26245319 PMC4588904

[B10] Fauve S, Heslot F (1983) Stochastic resonance in a bistable system. Phys Lett A 97:5–7. 10.1016/0375-9601(83)90086-5

[B11] Feldmann A, Whitt W (1998) Fitting mixtures of exponentials to long-tail distributions to analyze network performance models. Perf Eval 31:245–279. 10.1016/S0166-5316(97)00003-5

[B12] Friedrich R, Peinke J, Sahimi M, Reza Rahimi Tabar M (2011) Approaching complexity by stochastic methods: from biological systems to turbulence. Phys Rep 506:87–162. 10.1016/j.physrep.2011.05.003

[B13] Gammaitoni L, Hänggi P, Jung P, Marchesoni F (1998) Stochastic resonance. Rev Mod Phys 70:223–287. 10.1103/RevModPhys.70.223

[B14] Ghorbani M, Mehta M, Bruinsma R, Levine AJ (2012) Nonlinear-dynamics theory of up-down transitions in neocortical neural networks. Phys Rev E 85:021908. 10.1103/PhysRevE.85.021908 22463245

[B15] Giassi ACC, Duarte TT, Ellis W, Maler L (2012a) Organization of the gymnotiform fish pallium in relation to learning and memory: II. Extrinsic connections. J Comp Neur 520:3338–3368. 22430442 10.1002/cne.23109

[B16] Giassi ACC, Ellis W, Maler L (2012b) Organization of the gymnotiform fish pallium in relation to learning and memory: III. Intrinsic connections. J Comp Neur 520:3369–3394. 22434647 10.1002/cne.23108

[B17] Grillner S, Hellgren J, Ménard A, Saitoh K, Wikström MA (2005) Mechanisms for selection of basic motor programs – roles for the striatum and pallidum. Trends Neurosci 28:364–370. 10.1016/j.tins.2005.05.004 15935487

[B18] Harnos A, Horváth G, Lawrence AB, Vattay G (2000) Scaling and intermittency in animal behaviour. Physica A 286:312–320. 10.1016/S0378-4371(00)00332-0

[B19] Harvey-Girard E, Giassi ACC, Ellis W, Maler L (2013) Expression of the cannabinoid CB1 receptor in the gymnotiform fish brain and its implications for the organization of the teleost pallium. J Comp Neur 521:949–975. 10.1002/cne.23212 22886386

[B20] Hidalgo J, Seoane LF, Cortés JM, Muñoz MA (2012) Stochastic amplification of fluctuations in cortical up-states. PLoS One 7:e40710. 10.1371/journal.pone.0040710 22879879 PMC3413692

[B21] Hindriks R, Bijma F, van Dijk BW, van der Werf YD, van Someren EJW, van der Vaart AW (2011a) Dynamics underlying spontaneous human alpha oscillations: a data-driven approach. NeuroImage 57:440–451. 21558008 10.1016/j.neuroimage.2011.04.043

[B22] Hindriks R, Jansen R, Bijma F, Mansvelder HD, de Gunst MCM, van der Vaart AW (2011b) Unbiased estimation of Langevin dynamics from time series with application to hippocampal field potentials in vitro. Phys Rev E 84:021133. 10.1103/PhysRevE.84.021133 21928975

[B23] Holcman D, Tsodyks M (2006) The emergence of up and down states in cortical networks. PLoS Computat Biol 2:e23. 10.1371/journal.pcbi.0020023 16557293 PMC1409813

[B24] Huesa G, van den Pol AN, Finger TE (2005) Differential distribution of hypocretin (orexin) and melanin-concentrating hormone in the goldfish brain. J Comp Neur 488:476–491. 10.1002/cne.20610 15973685

[B25] Jens Prusseit KL (2007) Stochastic qualifiers of epileptic brain dynamics. Phys Rev Lett 98:138103. 10.1103/PhysRevLett.98.138103 17501243

[B26] Johnston DC (2006) Stretched exponential relaxation arising from a continuous sum of exponential decays. Phys Rev B 74:184430.

[B27] Jun JJ, Longtin A (2014) Long-term behavioral tracking of freely swimming weakly electric fish. J Vis Exp 85:e50962. 10.3791/50962 PMC4143086 24637642

[B28] Jun JJ, Longtin A, Maler L (2012) Precision measurement of electric organ discharge timing from freely moving weakly electric fish. J Neurophysiol 107:1996–2007. 22190625 10.1152/jn.00757.2011

[B29] Jun JJ, Longtin A, Maler L (2014) Enhanced sensory sampling precedes self-initiated locomotion in an electric fish. J Exp Biol 217:3615–3628. 10.1242/jeb.105502 25320268

[B30] Kagaya K, Takahata M (2010) Readiness discharge for spontaneous initiation of walking in crayfish. J Neurosci 30:1348–1362. 10.1523/JNEUROSCI.4885-09.2010 20107061 PMC6633775

[B31] Kaslin J, Nystedt JM, Ostergård M, Peitsaro N, Panula P (2004) The orexin/hypocretin system in zebrafish is connected to the aminergic and cholinergic systems. J Neurosci 24:2678–2689. 10.1523/JNEUROSCI.4908-03.2004 15028760 PMC6729510

[B32] Kornhuber HH, Deecke L (1965) Hirnpotentialänderungen bei Willkürbewegungen und passiven Bewegungen des Menschen: Bereitschaftspotential und reafferente Potentiale. Pflugers Arch 284:1–17. 10.1007/BF00412364 14341490

[B33] Kramer DL, McLaughlin RL (2001) The behavioral ecology of intermittent locomotion. Am Zool 41:137–153. 10.1093/icb/41.2.137

[B34] Lamouroux D, Lehnertz K (2009) Kernel-based regression of drift and diffusion coefficients of stochastic processes. Phys Lett A 373:3507–3512. 10.1016/j.physleta.2009.07.073

[B35] Lewicki MS (1998) A review of methods for spike sorting: the detection and classification of neural action potentials. Network 9:R53–R78. 10221571

[B36] Libet B, Gleason CA, Wright EW, Pearl DK (1983) Time of conscious intention to act in relation to onset of cerebral activity (readiness-potential). The unconscious initiation of a freely voluntary act. Brain 106:623–642. 10.1093/brain/106.3.623 6640273

[B37] Liebovitch LS, Tóth TI (1991) Distributions of activation energy barriers that produce stretched exponential probability distributions for the time spent in each state of the two state reaction A⇌B. Bull Math Biol 53:443–455.

[B38] Luevano J-R (2013) Statistical features of the stretched exponentials densities. J Phys Conf Ser 475:012008.

[B39] Maesani A, Ramdya P, Cruchet S, Gustafson K, Benton R, Floreano D (2015) Fluctuation-driven neural dynamics reproduce *Drosophila* locomotor patterns. PLoS Comput Biol 11:e1004577 10.1371/journal.pcbi.1004577 26600381 PMC4657918

[B40] Martí D, Deco G, Mattia M, Gigante G, Del Giudice P (2008) A fluctuation-driven mechanism for slow decision processes in reverberant networks. PLoS One 3:e2534 10.1371/journal.pone.0002534 18596965 PMC2432027

[B41] Mashanova A, Oliver TH, Jansen VAA (2010) Evidence for intermittency and a truncated power law from highly resolved aphid movement data. J R Soc Interface 7:199–208. 10.1098/rsif.2009.0121 19474077 PMC2839383

[B42] Maye A, Hsieh C-h, Sugihara G, Brembs B (2007) Order in spontaneous behavior. PLoS One 2:e443. 10.1371/journal.pone.0000443 17505542 PMC1865389

[B43] Mejias JF, Kappen HJ, Torres JJ (2010) Irregular dynamics in up and down cortical states. PLoS One 5:e13651. 10.1371/journal.pone.0013651 21079740 PMC2975677

[B44] Murakami M, Vicente MI, Costa GM, Mainen ZF (2014) Neural antecedents of self-initiated actions in secondary motor cortex. Nat Neurosci 17:1574–1582. 10.1038/nn.3826 25262496

[B45] Patankar S (1980) Numerical heat transfer and fluid flow. Boca Raton: CRC Press.

[B46] Pereira AC, Rodríguez-Cattáneo A, Caputi AA (2014) The slow pathway in the electrosensory lobe of *Gymnotus omarorum*: field potentials and unitary activity. J Physiol Paris 108:71–83. 10.1016/j.jphysparis.2014.07.005 25088503

[B47] Pikovsky AS, Kurths J (1997) Coherence resonance in a noise-driven excitable system. Phys Rev Lett 78:775–778. 10.1103/PhysRevLett.78.775

[B48] Prober DA, Rihel J, Onah AA, Sung R-J, Schier AF (2006) Hypocretin/orexin overexpression induces an insomnia-like phenotype in zebrafish. J Neurosci 26:13400–13410. 10.1523/JNEUROSCI.4332-06.2006 17182791 PMC6675014

[B49] Proekt A, Banavar JR, Maritan A, Pfaff DW (2012) Scale invariance in the dynamics of spontaneous behavior. Proc Natl Acad Sci USA 109:10564–10569. 10.1073/pnas.1206894109 22679281 PMC3387096

[B50] Roxin A, Ledberg A (2008) Neurobiological models of two-choice decision making can be reduced to a one-dimensional nonlinear diffusion equation. PLoS Comput Biol 4:e1000046. 10.1371/journal.pcbi.1000046 18369436 PMC2268007

[B51] Schurger A, Sitt JD, Dehaene S (2012) An accumulator model for spontaneous neural activity prior to self-initiated movement. Proc Natl Acad Sci USA 109:E2904–E2913. 10.1073/pnas.1210467109 22869750 PMC3479453

[B52] Shadlen MN, Kiani R (2013) Decision making as a window on cognition. Neuron 80:791–806. 10.1016/j.neuron.2013.10.047 24183028 PMC3852636

[B53] Stephens GJ, Osborne LC, Bialek W (2011) Searching for simplicity in the analysis of neurons and behavior. Proc Natl Acad Sci USA 108:15565–15571. 10.1073/pnas.1010868108 21383186 PMC3176616

[B54] Trinh A-T, Harvey-Girard E, Teixeira F, Maler L (2016) Cryptic laminar and columnar organization in the dorsolateral pallium of a weakly electric fish. J Comp Neur 524:408–428. 10.1002/cne.23874 26234725

[B55] Tu Y, Grinstein G (2005) How white noise generates power-law switching in bacterial flagellar motors. Phys Rev Lett 94:208101. 10.1103/PhysRevLett.94.208101 16090291

[B56] Wang X-J (2002) Probabilistic decision making by slow reverberation in cortical circuits. Neuron 36:955–968. 12467598 10.1016/s0896-6273(02)01092-9

[B57] Wang X-J (2008) Decision making in recurrent neuronal circuits. Neuron 60:215–234. 10.1016/j.neuron.2008.09.034 18957215 PMC2710297

[B58] Wilson CJ, Kawaguchi Y (1996) The origins of two-state spontaneous membrane potential fluctuations of neostriatal spiny neurons. J Neurosci 16:2397–2410. 8601819 10.1523/JNEUROSCI.16-07-02397.1996 PMC6578540

[B59] Wong CJ (1997) Afferent and efferent connections of the diencephalic prepacemaker nucleus in the weakly electric fish, *Eigenmannia virescens*: interactions between the electromotor system and the neuroendocrine axis. J Comp Neur 383:18–41. 10.1002/(SICI)1096-9861(19970623)383:1<18::AID-CNE2>3.0.CO;2-O 9184983

[B60] Woods IG, Schoppik D, Shi VJ, Zimmerman S, Coleman HA, Greenwood J, Soucy ER, Schier AF (2014) Neuropeptidergic signaling partitions arousal behaviors in zebrafish. J Neurosci 34:3142–3160. 10.1523/JNEUROSCI.3529-13.2014 24573274 PMC3935080

[B61] Yamamoto T, Maler L (1992) Organization of galanin-like immunoreactive neuronal systems in weakly electric fish (Apteronotus leptorhynchus). J Chem Neuroanat 5:19–38. 1376606 10.1016/0891-0618(92)90031-k

